# RAB-6.1 and RAB-6.2 Promote Retrograde Transport in *C*. *elegans*

**DOI:** 10.1371/journal.pone.0149314

**Published:** 2016-02-18

**Authors:** Donglei Zhang, Jyoti Dubey, Sandhya P. Koushika, Christopher Rongo

**Affiliations:** 1 The Waksman Institute, Department of Genetics, Rutgers The State University of New Jersey, Piscataway, New Jersey, United States of America; 2 Department of Biological Sciences, Tata Institute of Fundamental Research, Colaba, Mumbai, India; 3 Institute for Stem Cell Biology and Regenerative Medicine (InStem), Bangalore, India; 4 Manipal University, Karnataka, India; Institut Curie, FRANCE

## Abstract

Retrograde transport is a critical mechanism for recycling certain membrane cargo. Following endocytosis from the plasma membrane, retrograde cargo is moved from early endosomes to Golgi followed by transport (recycling) back to the plasma membrane. The complete molecular and cellular mechanisms of retrograde transport remain unclear. The small GTPase RAB-6.2 mediates the retrograde recycling of the AMPA-type glutamate receptor (AMPAR) subunit GLR-1 in *C*. *elegans* neurons. Here we show that RAB-6.2 and a close paralog, RAB-6.1, together regulate retrograde transport in both neurons and non-neuronal tissue. Mutants for *rab-6*.*1* or *rab-6*.*2* fail to recycle GLR-1 receptors, resulting in GLR-1 turnover and behavioral defects indicative of diminished GLR-1 function. Loss of both *rab-6*.*1* and *rab-6*.*2* results in an additive effect on GLR-1 retrograde recycling, indicating that these two *C*. *elegans* Rab6 isoforms have overlapping functions. MIG-14 (Wntless) protein, which undergoes retrograde recycling, undergoes a similar degradation in intestinal epithelia in both *rab-6*.*1* and *rab-6*.*2* mutants, suggesting a broader role for these proteins in retrograde transport. Surprisingly, MIG-14 is localized to separate, spatially segregated endosomal compartments in *rab-6*.*1* mutants compared to *rab-6*.*2* mutants. Our results indicate that RAB-6.1 and RAB-6.2 have partially redundant functions in overall retrograde transport, but also have their own unique cellular- and subcellular functions.

## Introduction

Cells direct their subcellular organization through the regulated trafficking of lipids and membrane-bound proteins to specific membrane compartments. Membrane trafficking in turn is organized by Rab proteins, which are members of the Ras small GTPase superfamily [1,2]. Rabs act as master regulators of transport between membrane compartments within cells, recruiting multiple effector molecules that recognize cargo, promote membrane fission and fusion, alter lipid composition, mediate transport along cytoskeletal elements, and regulate other Rabs. How multiple Rabs work together to regulate specific membrane trafficking pathways remains an open question.

Individual Rab family members appear to direct traffic between membranes of specific subcellular structures, and the large number of Rab family members reflects the numerous and diverse transport pathways that exist within cells. In mammals, more than 70 Rab GTPases have been identified [1]. In the nematode *C*. *elegans*, which has been employed as a powerful genetic model system for studying membrane trafficking in a multicellular context, about 30 Rabs have been identified based on sequence similarity with their mammalian orthologs [3,4]. Most Rabs are ubiquitously expressed, generally regulating the same corresponding trafficking steps in different tissues, and their abundance in specific tissues usually reflects the levels of corresponding trafficking steps that needed to be regulated within those tissues. However, some Rabs are involved in specialized cellular functions and are expressed in particular cells. For example, regulated neurotransmitter release at neuronal active zones is specifically controlled by Rab3A [5]. An understanding of the dedicated function of each Rab family member should shed light on the strategy by which cells organize their membrane compartments.

Multiple Rab GTPase family members are further differentiated into separate isoform paralogs. In mammals, Rab1, Rab3, Rab4, Rab5, Rab6, Rab7, Rab8, Rab11, Rab22, Rab27, Rab33, and Rab39 have been described as having different isoforms [6–10]. The precise function of these different isoforms has largely remained uncharacterized. Some isoforms are abundant in different tissues or localize to different intracellular compartments to play distinct roles in a single cell. For example, mammalian Rab3A and Rab3C are primarily expressed in neurons and neuroendocrine cells [11,12], whereas Rab3B is expressed in adipocytes [13]. Moreover, the Rab3A, B, C and D isoforms have different subcellular distributions in insulin-secreting cells [14]. In addition to conducting divergent functions, Rab isoforms can also function redundantly. For instance, mammalian Rab1A and Rab1B have the same function in the regulation of ER-to-Golgi transport [15].

The Rab6 subfamily is of particular interest due to its roles in Golgi-ER trafficking and retrograde recycling from early endosomes to Golgi. In mammals, Rab6 has four isoforms, including Rab6A, A’, B, and C. Rab6A and Rab6A’ are generated by alternative splicing of the same gene [16], whereas Rab6B is encoded by a totally separate gene [17]. In addition, primates possess a separate Rab6C gene, which appears to be a retrogene that originally derived from a Rab6A’ transcript [18]. Rab6A appears to regulate retrograde transport from the Golgi to the ER, as constitutively active Rab6A, induced through a GTPase-deficient mutation, induces the redistribution of Golgi resident proteins into the ER [19]. However, although Rab6A and Rab6A’ are both expressed ubiquitously and only differ in three amino acid residues, Rab6A’ does not regulate the same transport step as Rab6A. Rather, Rab6A and Rab6A’ regulate sequential retrograde trafficking steps, with Rab6A regulating COPI-independent retrograde trafficking from the Golgi to ER, and Rab6A’ regulating retrograde trafficking from endosomes to Golgi [16,20]. Unlike Rab6A and A’, Rab6B displays a tissue specific expression pattern and is predominantly expressed in the brain [17]. Like Rab6A, Rab6B is found at the Golgi apparatus. In addition, Rab6B interacts with some of the same Rab6A effector molecules [17]. The primate specific Rab6C is only expressed in a limited number of human tissues and is localized to the centrosome. Overexpression of Rab6C causes G1 arrest, indicating a function for Rab6C in the cell cycle [18].

The divergence of the Rab6 subfamily might be an evolutionarily old event, as in *C*. *elegans* there are two Rab6 isoforms: RAB-6.1 and RAB-6.2. Whereas the function of RAB-6.1 remains unknown, we and others previously showed that RAB-6.2 regulates the transport of retrograde cargo, including β1 integrin and AMPA-type glutamate receptors (AMPARs) [21,22]. AMPARs are of particular interest, as they mediate excitatory synaptic transmission in the brain, and the regulated trafficking and recycling of AMPARs at the synapse is a pivotal mechanism by which neurons regulate synaptic strength during learning and memory [23,24]. The *C*. *elegans* AMPAR subunit GLR-1, like its mammalian counterparts, also undergoes regulated trafficking and recycling. GLR-1 acts in the command interneurons where it transduces synaptic input from nose-touch mechanosensory neurons and governs overall locomotory behavior [25–28], including spontaneous reversals in the direction of locomotion [29]. Mutants that lack AMPAR function or fail to transport and maintain AMPARs at synapses have a depressed frequency of spontaneous reversals; thus, these behaviors correlate with AMPAR synaptic abundance [30–32]. In the absence of *rab-6*.*2*, GLR-1 receptors fail to be recycled to synapses and are shunted to late endosomes for turnover [21]. In addition, expression of a constitutively active *rab-6*.*2* is sufficient to drive GLR-1 receptors out of dendrites and into cell body Golgi in a retrograde fashion. GLR-1 recycling is also mediated by the retromer complex, which is comprised of sorting nexins (Vps5/SNX1/2) and the VPS26-VPS29-VPS35 subcomplex, and is associated with the J-domain protein RME-8 [21,33–36]. How the function of the Rab6 GTPases is integrated with that of the retromer is unclear [16,20,37].

Given the sequence similarity of RAB-6.1 to RAB-6.2, we hypothesized that RAB-6.1 might also contribute to regulating GLR-1 recycling. Here we show that RAB-6.1 and RAB-6.2 act in a partially redundant fashion to promote GLR-1 recycling in neurons and GLR-1-mediated behavior. Loss of both *rab-6*.*1* and *rab-6*.*2* results in lethality, indicating that these two *C*. *elegans* Rab6 isoforms have overlapping functions in other tissues. MIG-14 (Wntless) protein, which undergoes retrograde recycling in intestinal epithelia [36,38–43], also requires RAB-6.1 and RAB-6.2 for proper recycling, suggesting a broader role for these proteins in retrograde transport. We also find that MIG-14 is localized to separate, spatially segregated endosomal compartments in *rab-6*.*1* mutants compared to *rab-6*.*2* mutants. We propose that RAB-6.1 and RAB-6.2 have partially redundant functions in overall retrograde transport, but also have their own unique cellular- and subcellular functions.

## Results

### RAB-6.1 regulates GLR-1 trafficking and GLR-1-mediated behavior

**Fig 1:**
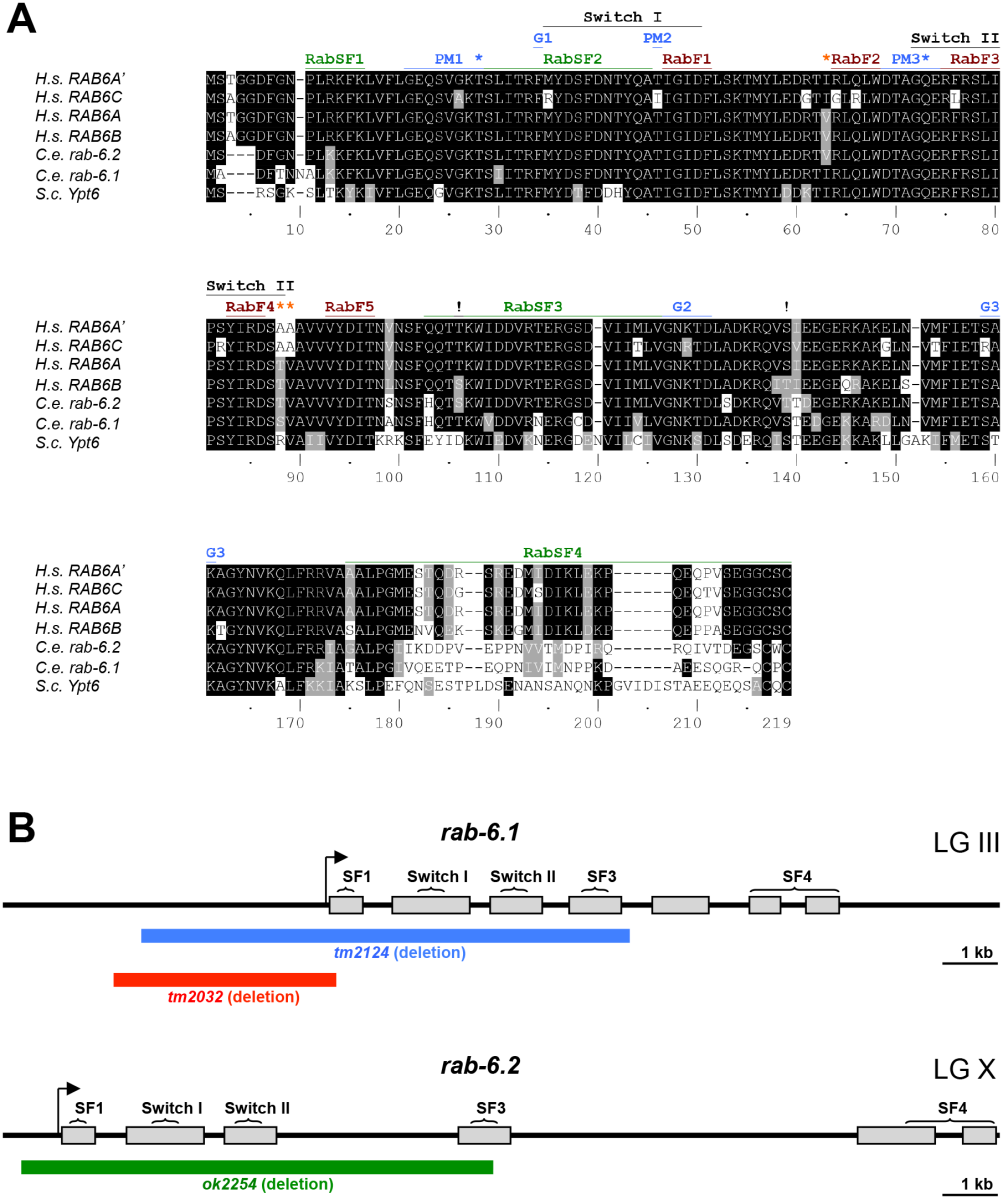
Gene structure for *rab-6*.*1* and *rab-6*.*2*. (A) Sequence alignments based on ClustalW for four human (H.s.) Rab6 isoforms, the yeast (S.c.) isoform Ypt6, and the two *C*. *elegans* (C.e.) Rab6 isoforms. Conservation of identical and similar amino acids are indicated by black and gray highlighting, respectively. “RabF” refers to Rab family specific regions. “RabSF” refers to Rab subfamily specific regions. “PM” refers to phosphate/magnesium binding residues. “G” refers to guanine nucleotide binding regions. “!” refers to amino acids that differentiate the Rab6A/RAB-6.1 subfamily from the Rab6B/RAB-6.2 subfamily. Orange asterisks indicate the three residues that differ between RAB6A and RAB6A’. Blue asterisks indicate the sites commonly mutated to generate GTP-locked and GDP-locked mutant proteins. The Switch I and Switch II regions are also indicated. Accession numbers: CAG46781.1 (Rab6A); AAF73841.1 (Rab6A’); AAF61637.1 (Rab6B); CAG38500.1 (Rab6C); CAA77590.1 (RAB-6.1); C*CD74453*.*1 (RAB-6*.*2); and Q99260*.*1 (Ypt6)*. (B) Genomic organization for *rab-6*.*1* and *rab-6*.*2*. Gray boxes indicate exons. The arrow indicates the start of transcription based on EST alignments with SL1 leader sequences. The brackets indicate the regions of genomic DNA that encode the indicated protein domains. The blue, red, and green lines indicate the regions of genomic DNA deleted in the *tm2124*, *tm2032*, and *ok2254* mutants, respectively. The chromosomal linkage groups (LG) for each gene are indicated.

As in the human genome, where there are multiple Rab6 subfamily isoforms, there are two Rab6 subfamily members in the *C*. *elegans* genome: RAB-6.1 and RAB-6.2. Pairwise sequence alignment analysis reveals that these two isoforms share 90% amino acid conservation, similar to the conservation observed between human Rab6 isoforms. The human Rab6 isoforms differ from each other at several key residues in the Switch II domain and the third Rab superfamily (RabSF) domain (Fig 1A and S1 Text). For example, Rab6A and Rab6B contain valine at position 63, whereas Rab6A’ and Rab6C contain isoleucine. Rab6B differs from Rab6A by using serine at position 106 and threonine at position 139, whereas Rab6A uses threonine and serine at those positions, respectively. We found that *C*. *elegans* RAB-6.1 contains valine at position 63 (similar to human Rab6A’ and Rab6C), whereas RAB-6.2 contains isoleucine (similar to human Rab6A and Rab6B). We also found that *C*. *elegans* RAB-6.1 contains threonine and serine at positions 106 and 139, respectively (similar to Rab6A, Rab6A’, and Rab6C), whereas RAB-6.2 contains serine and threonine at those positions (similar to Rab6B). *Saccharomyces cerevisiae* has a single Rab6 in its genome, Ypt6, which contains isoleucine, aspartate, and threonine at residues 63, 106, and 139, making Ypt6 more similar to Rab6A’, Rab6C, and RAB-6.1 (and to a lesser degree, Rab6A) than it is to Rab6B and RAB-6.2 (Fig 1A and S1 Text). Our sequence analysis suggests that RAB-6.1 is a potential *C*. *elegans* ortholog of Rab6A’, Rab6A, and/or Rab6C, whereas RAB-6.2 is a potential *C*. *elegans* ortholog of Rab6B. We suggest that these isoform subgroups diverged prior to the vertebrate-invertebrate split, and that the Rab6A, Rab6A’, Rab6C, RAB-6.1, Ypt6 subgroup more closely resembles the ancestral Rab6 founder.

We previously demonstrated that RAB-6.2 promotes the retrograde recycling of GLR-1 AMPARs to synapses [21]. Based on their sequence similarity, we hypothesized that RAB-6.1 might function redundantly with RAB-6.2 to regulate GLR-1 trafficking in *C*. *elegans*. To study functions of RAB-6.1 on GLR-1 transport, we obtained two mutant strains from the *C*. *elegans* Japanese Knockout Consortium that carry deletions of *rab-6*.*1* sequences: *rab-6*.*1(tm2032)* and *rab-6*.*1(tm2124)*. The *tm2032* deletion removes part of the *rab-6*.*1* promoter, its 5’ UTR, its start codon, and a small segment of sequence encoding the N-terminus of the RAB-6.1 protein (Fig 1B). The *tm2124 d*eletion also removes part of the *rab-6*.*1* promoter, as well as sequences encoding RabSF1, Switch I, Switch II, and RabSF3 domains. Both of these two mutants are likely nulls of *rab-6*.*1*. However, we could not maintain homozygous hermaphrodite strains for these mutants even after several rounds of outcrossing. To analyze the *rab-6*.*1* mutant phenotype more easily, we balanced these two alleles by generating *rab-6*.*1(tm2032)/hT2(I*, *III)* and *rab-6*.*1(tm2124)/hT2(I*, *III)* strains. We found that both homozygous *rab-6*.*1(tm2032)* and *rab-6*.*1(tm2124)* can develop through the adult stage without any delays or visible morphological defects. However, fertilized embryos did not develop in the gonad, indicating that *rab-6*.*1* homozygous mutant hermaphrodite animals are sterile. Interestingly, the sterility phenotype was fully rescued when *rab-6*.*1* homozygous hermaphrodites were mated to wild-type males, suggesting that the sterile phenotype is due to a defect in hermaphrodite sperm and not due to a defect in oocytes. Because *rab-6*.*1(tm2124)* contains a deletion that covers the most extensive region of the *rab-6*.*1* gene, we have only used *rab-6*.*1(tm2124)* to study the functions of RAB-6.1 in the following experiments.

**Fig 2:**
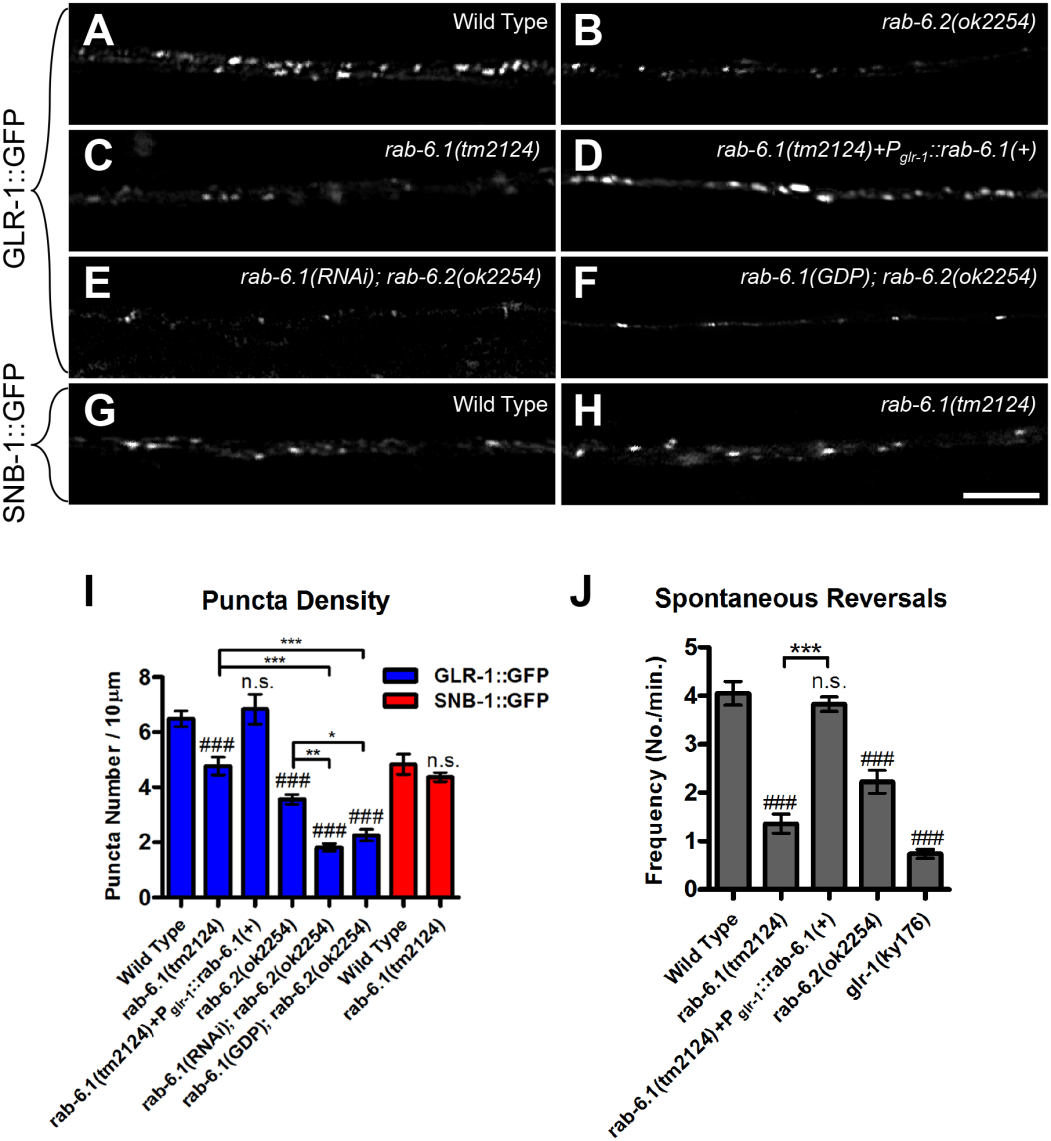
RAB-6.1 regulates GLR-1 synaptic localization. GLR-1::GFP fluorescence along ventral cord dendrites is indicated in (A) wild-type animals, (B) *rab-6*.*2(ok2254)* mutants, (C) *rab-6*.*1(tm2124)* mutants, (D) *rab-6*.*1(tm2124)* mutants expressing a wild-type *rab-6*.*1* cDNA from the *glr-1* promoter, (E) *rab-6*.*1(ok2254)* mutants expressing a *rab-6*.*1* hairpin RNA, and (F) *rab-6*.*2(ok2254)* mutants expressing a dominant negative RAB-6.1(GDP) protein. Fluorescence from the synaptic vesicle protein SNB-1(synaptobrevin)::GFP is indicated in (G) wild-type animals and (H) *rab-6*.*1(tm2124)* mutants. (I) The mean density of fluorescent puncta for the given genotype and fluorescent reporter (blue bars, GLR-1::GFP; red bars, SNB-1::GFP). (J) The mean spontaneous reversal frequency for the indicated genotypes. Bar, 5 μm. Error bars are SEM. N = 15–35 animals. ANOVA with Dunnett’s multiple comparison to wild type (###, p<0.001) or Bonferroni Multiple Comparison test (*p<0.05; **p<0.01; ***p<0.001). “n.s.” indicates non-significance.

To determine whether RAB-6.1 regulates GLR-1 trafficking, we introduced *nuIs25[glr-1*::*gfp]* into outcrossed *rab-6*.*1(tm2124)/hT2(I*, *III)* mutants. The *nuIs25[glr-1*::*gfp]* transgenic strain expresses a rescuing GLR-1::GFP chimeric receptor that is localized to the postsynaptic face of synapses [30,44]. Individual GLR-1::GFP-containing synapses can be observed as small (0.5–0.7 micron) puncta along the ventral cord dendrites in wild-type young adult animals (Fig 2A). To repeat our original findings, we re-examined *rab-6*.*2(ok2254)* mutants, which lack the start of transcription, the start codon, and both switch domains of this small GTPase, making it a likely molecular null (Fig 1B). We observed a decrease in the number and fluorescence intensity of GLR-1::GFP puncta in *rab-6*.*2(ok2254)* mutants compared to wild type (Fig 2B and 2I). We next observed the subcellular localization of GLR-1::GFP in *rab-6*.*1(tm2124)* homozygotes and found a significant decrease in the number of GLR-1::GFP puncta along the ventral nerve cord (Fig 2C and 2I), although not to the same extent as we observed in *rab-6*.*2* mutants. We also examined the localization of a presynaptic protein, SNB-1 (synaptobrevin) [45], and observed no difference in GFP-labeled SNB-1 puncta in mutants compared to wild type (Fig 2G, 2H and 2I), indicating that the defects in GLR-1 subcellular localization are not due to gross defects in synapse formation along GLR-1-expressing interneurons. Our results indicate that RAB-6.1, like RAB-6.2, regulates the subcellular localization of GLR-1 receptors.

A decrease in synaptic GLR-1::GFP should result in a decrease in GLR-1 function. GLR-1 AMPARs act in the command interneurons to promote spontaneous reversals in locomotion as nematodes forage for food in their environment. Thus, GLR-1 function can be evaluated by calculating the spontaneous reversal frequency of individual animals because mutants with defects in GLR-1 gating properties or GLR-1 synaptic localization have a lower frequency of spontaneous reversals [28,30,46]. We found that the spontaneous reversal rate (Fig 2J) of *rab-6*.*1* mutants was significantly lower than that in wild type, indicating that a loss of endogenous GLR-1 function accompanies the drop in synaptic GLR-1 in these mutants. RAB-6.1 promotes GLR-1 function as well as GLR-1 trafficking, and while there are more GLR-1 puncta remaining in *rab-6*.*1* mutants compared to *rab-6*.*2* mutants, the remaining localized GLR-1 in *rab-6*.*1* mutants does not appear to be sufficient to mediate additional reversal behavior.

### RAB-6.1 is expressed in GLR-1-expressing neurons

**Fig 3:**
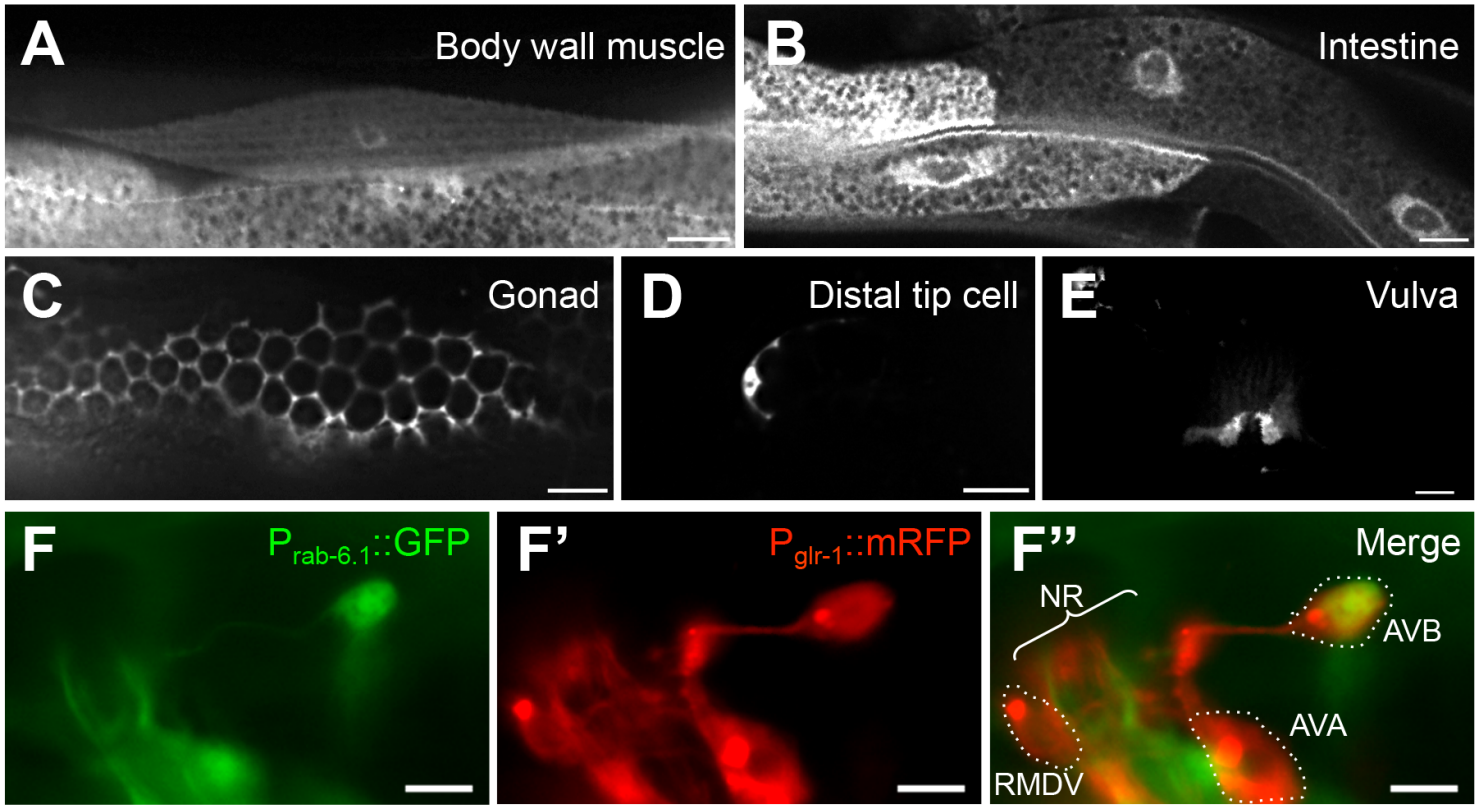
RAB-6.1 is broadly expressed in multiple tissues. Fluorescence from *P*_*rab-6*.*1*_::*GFP* in (A) body wall muscles, (B) intestinal epithelia, (C) the gonad, (D) the distal tip cells, and (E) the vulva. Fluorescence from (F) *P*_*rab-6*.*1*_::*GFP* and (F’) *P*_*glr-1*_::*mRFP*, with (E”) images merged. The neuron cell bodies for RMDV, AVA, and AVB are outlined. NR indicated the nerve ring bundle of neurites. Bar, 5 μm.

To determine the expression pattern of RAB-6.1, we generated transgenes containing the *rab-6*.*1* promoter (3 kb of upstream from the start codon) and the complete genomic sequences (including all exons and introns) fused to C-terminal GFP sequences. We introduced the *rab-6*.*1*::*gfp* reporter into wild type animals and found that RAB-6.1 is broadly expressed in most tissues, with a large degree of overlapping expression with RAB-6.2 (Fig 3). RAB-6.1::GFP is highly expressed in body wall muscle (Fig 3A), intestine (Fig 3B), somatic gonad (Fig 3C), distal tip cells (Fig 3D), vulva (Fig 3E), and neurons (Fig 3F). To test whether RAB-6.1 is expressed in the GLR-1-expressing command interneurons, we introduced the *rab-6*.*1*::*gfp* reporter into a strain carrying a *P*_*glr-1*_::*mRFP* reporter, which illuminates the command interneurons with mRFP [32]. We found that RAB-6.1 is expressed in multiple GLR-1-expressing neurons (Fig 3F and 3F”), including AVB, AVD, RIG, and PVC, but not AVA or RMDV.

To determine whether RAB-6.1 acts in the command interneurons to regulate GLR-1 cell autonomously, we generated a rescuing transgene containing *glr-1* promoter sequences fused to *rab-6*.*1* cDNA sequences. We introduced this *P*_*glr-1*_::*rab-6*.*1(+)* transgene into *rab-6*.*1* mutants that also express GLR-1::GFP. We found that *P*_*glr-1*_::*rab-6*.*1(+)* completely rescued the defects in GLR-1::GFP puncta number (Fig 2D and 2I), as well as the defects in spontaneous reversal frequency (Fig 2J), indicating that RAB-6.1 functions cell autonomously to regulate GLR-1 localization and function.

### RAB-6.1 and RAB-6.2 have an additive effect on GLR-1 localization

As RAB-6.1 and RAB-6.2 proteins are similar in sequence (Fig 1A), and given that mutants for either of their corresponding genes have a partial defect in GLR-1 localization (Fig 2I), we reasoned that the two genes might have overlapping, partially redundant functions in regulating GLR-1 localization. We set out to test this possibility by introducing the *rab-6*.*1(tm2124*) mutation into the *rab-6*.*2(ok2254)* homozygous genetic background. We generated animals that were heterozygous for both mutations. However, despite employing several different crossing strategies, we could not obtain nematodes that were heterozygous for *rab-6*.*1(tm2124)* and homozygous for *rab-6*.*2(ok2254)*, nor nematodes that were homozygous for *rab-6*.*1(tm2124)* and heterozygous for *rab-6*.*2(ok2254)*, suggesting a synthetic lethal genetic interaction. As an alternative approach, we grew *rab-6*.*1(tm2124)/hT2* balanced nematodes (young L1 larvae) on feeding RNAi bacteria for *rab-6*.*2* until adulthood and examined their progeny. While we could obtain viable *rab-6*.*1(tm2124)/hT2* heterozygous progeny on *rab-6*.*2(RNAi)* knockdown media, we could not obtain *rab-6*.*1* homozygotes. We also grew *rab-6*.*2(ok2254)* L1-stage homozygotes on *rab-6*.*1(RNAi)* knockdown media, but recovered no viable progeny. Taken together, our results suggest that animals that are impaired for both Rab6 proteins are inviable. The synthetic lethality precluded direct analysis of GLR-1 trafficking in the double mutants, but did support the idea that there is partial functional redundancy between RAB-6.1 and RAB-6.2.

We used two approaches to get around this problem and examine GLR-1 in the absence of both Rab6 proteins. First, we generated an RNAi knockdown transgene that expresses a hairpin RNA for *rab-6*.*1* from the *glr-1* promoter (i.e., genetically encoded, tissue specific RNAi). We introduced *P*_*glr-1*_::*rab-6*.*1(RNAi)* into *rab-6*.*2* mutants and observed a dramatic decrease in the number of GLR-1::GFP puncta compared to either *rab-6*.*1* single mutants or *rab-6*.*2* single mutants (Fig 2E and 2I). As a second approach, we generated a transgene that expresses a RAB-6.1 protein with the mutation T25N, resulting in a dominant negative, GDP-locked RAB-6.1. We restricted expression of this mutant RAB-6.1(GDP) to postembryonic command interneurons by using the *glr-1* promoter. We introduced *P*_*glr-1*_::*rab-6*.*1(GDP)* into *rab-6*.*2* mutants and observed a significant decrease in the number of GLR-1::GFP puncta compared to *rab-6*.*1* mutants or *rab-6*.*2* mutants alone (Fig 2F and 2I). These data indicate that the loss of both Rab6 genes in *C*. *elegans* gives a stronger phenotype than the loss of either one alone, and that RAB-6.1 and RAB-6.2 have partially but not completely overlapping functions with respect to GLR-1 trafficking.

### RAB-6.1 regulates GLR-1 trafficking at a step after endocytosis

What is the cell biological mechanism by which Rab6 regulates GLR-1 localization in neurons? RAB-6.2 promotes the recycling of GLR-1, and Rab6 proteins are known to facilitate the retrograde transport of endocytosed cargo from endosomes to Golgi [20,21,34]. However, based on studies of Rab6 in the dynamic transport of vesicles between the Golgi and the cell periphery, Rab6 proteins can also target exocytotic carrier vesicles and regulate secretory transport [47–49]. If RAB-6.1 regulates GLR-1 post-endocytic recycling rather than secretion (similar to what has been observed for RAB-6.2), then the defects in GLR-1 localization observed in *rab-6*.*1* mutants should be dependent on the preceding endocytosis of GLR-1 receptors. We therefore tested whether endocytosis is required for the receptor turnover that we observed in *rab-6*.*1* mutants, examining whether mutations that block GLR-1 endocytosis also suppress the GLR-1 trafficking defects in *rab-6*.*1* mutants.

**Fig 4:**
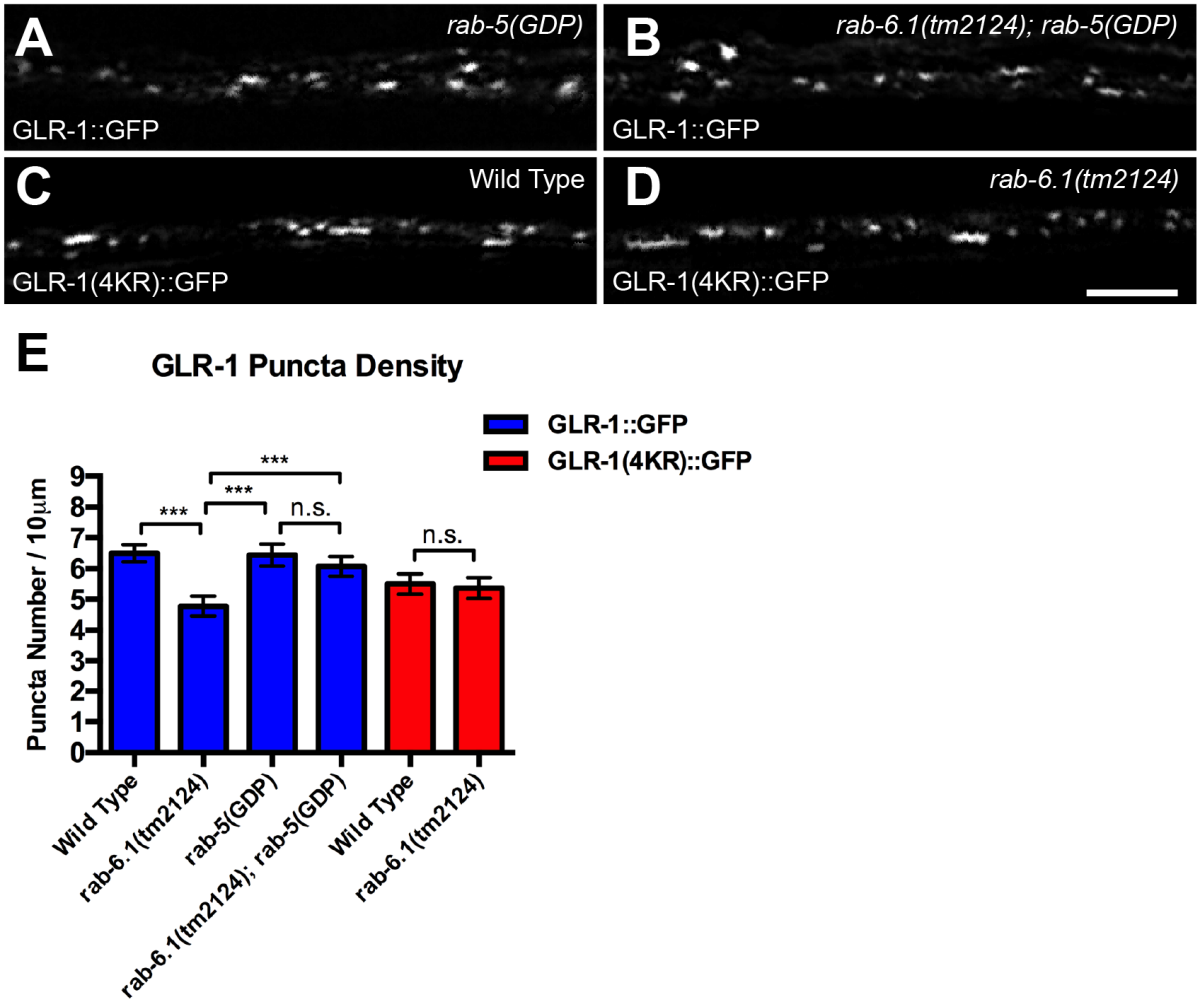
RAB-6.1 functions downstream of GLR-1 endocytosis. GLR-1::GFP fluorescence in (A) wild-type animals that express a dominant negative RAB-5(GDP) or (B) *rab-6*.*1(tm2124)* mutants that express RAB-5(GDP). The fluorescence of GLR-1(4KR)::GFP in (C) wild-type animals or (D) *rab-6*.*1(tm2124)* mutants. (E) The mean density of fluorescent puncta for the given genotype and reporter (blue bars, GLR-1::GFP; red bars, GLR-1(4KR)::GFP). Bar, 5 μm. Error bars are SEM. N = 20–30 animals. ANOVA with Bonferroni Multiple Comparison test. “n.s.” indicates non-significance.

We blocked GLR-1 endocytosis using two approaches. First, we altered Rab5 activity, as the Rab5 GTPase is a key regulator that controls clathrin-dependent endocytosis [50]. Expression of a dominant negative RAB-5(GDP) via the *glr-1* promoter dramatically blocks GLR-1 endocytosis [51]. We compared GLR-1::GFP localization in *rab-6*.*1* mutants to that in *rab-6*.*1; P*_*glr-1*_::*rab-5(GDP)* double mutants and found that the numbers of GLR-1 puncta are restored to a similar level observed in wild type (Fig 4A, 4B and 4E). Second, we altered GLR-1 monoubiquitination, which directly triggers receptor endocytosis [30]. We employed a *glr-1(4kr)*::*gfp* transgene that expresses a GLR-1::GFP in which the ubiquitinated lysines on the GLR-1 C-terminus are mutated to arginines, thereby precluding ubiquitination of GLR-1 and depressing receptor endocytosis and GLR-1 turnover [30]. We analyzed GLR-1(4KR)::GFP localization in *rab-6*.*1* mutants and found that the number of GLR-1(4KR)::GFP puncta is not altered in *rab-6*.*1* mutants compared to wild type (Fig 4C–4E). Our results indicate that endocytosis is required for the decreased levels of receptor observed in *rab-6*.*1* mutants, suggesting that RAB-6.1 regulates GLR-1 trafficking at a step after endocytosis.

### RAB-6.1 regulates the exit of GLR-1 from endosomes and GLR-1 turnover

**Fig 5:**
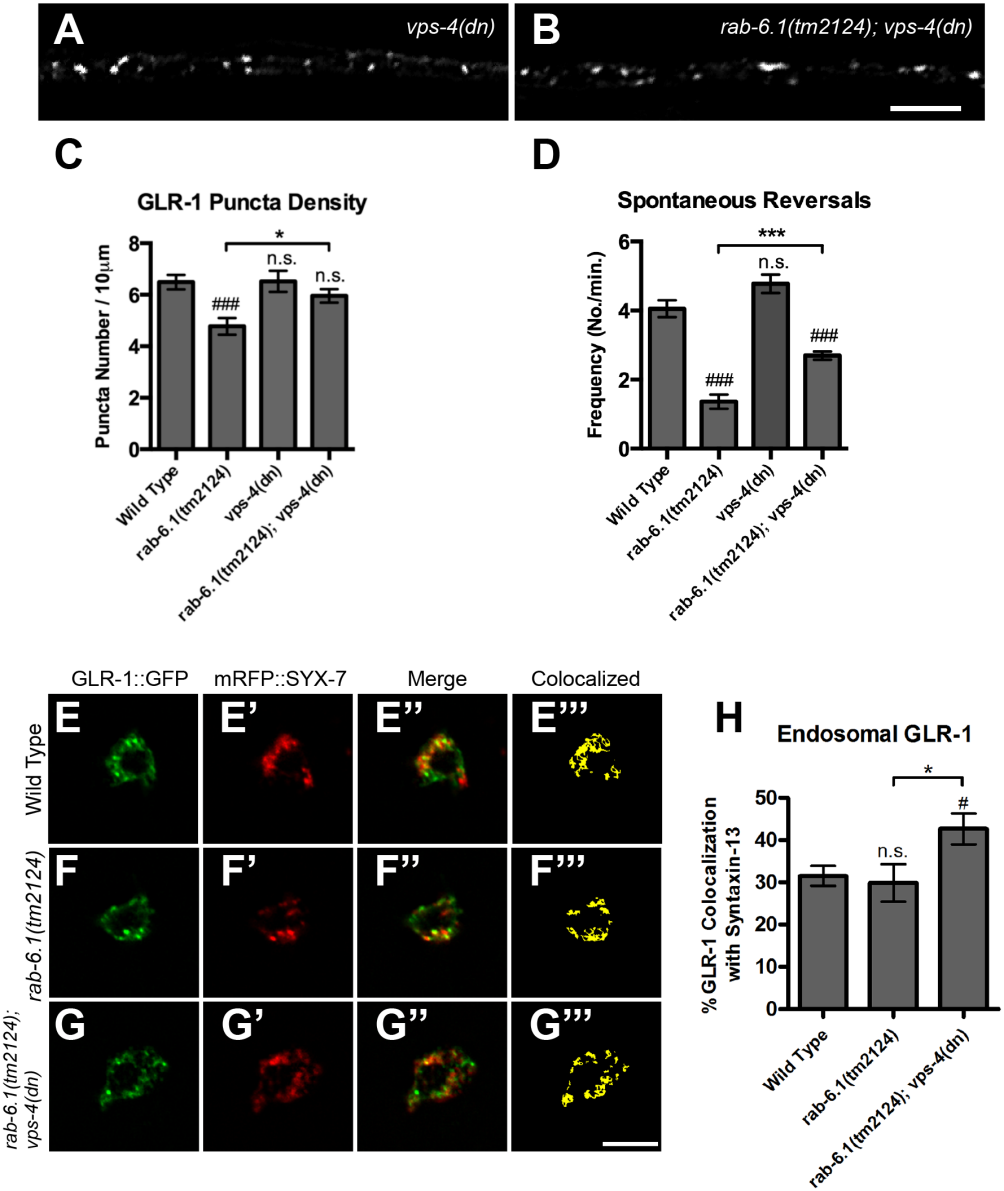
RAB-6.1 promotes GLR-1 recycling from endosomes and prevents turnover. GLR-1::GFP fluorescence observed in ventral cord dendrites of animals that express a dominant negative (dn) VPS-4 ESCRT subunit in (A) wild type or (B) *rab-6*.*1(tm2124)*. The (C) mean density of GLR-1 puncta and (D) spontaneous reversal frequency for the indicated genotype. (E, F, G) GLR-1::GFP and (E’, F’, G’) mRFP::SYX-7 (Syntaxin-13) fluorescence in neuron cell bodies from (E-E”‘) wild-type animals, (F-F”‘) *rab-6*.*1(tm2124)* mutants, and (G-G”‘) *rab-6*.*1(tm2124); vps-4(dn)* double mutants. Merged images are shown in E”, F”, and G”. Binary masks (E”‘, F”‘, G”‘) indicate colocalization by highlighting pixels with matching intensity values. (H) The mean percent of GLR-1::GFP, colocalized with endosomal marker mRFP::SYX-7, normalized to total GLR-1::GFP in cell bodies. Bar, 5 μm. Error bars are SEM. N = 20–35 animals. ANOVA with Dunnett’s multiple comparison to wild type (#p<0.05; ###p<0.001) or Bonferroni Multiple Comparison test (*p<0.05; ***p<0.001). “n.s.” indicates non-significance.

The decrease in GLR-1::GFP puncta that accompanies the GLR-1 recycling defects observed in *rab-6*.*1* mutants suggests that GLR-1 might be improperly sorted from early endosomes to multivesicular bodies (MVBs), late endosomes, and eventually lysosomes via ESCRT-mediated transport in these mutants [36,38–43]. We tested whether GLR-1 was degraded in *rab-6*.*1* mutants by blocking ESCRT-mediated transport and observing whether GLR-1 would accumulate in endosomes. The VPS-4 AAA ATPase facilitates the movement of endocytosed cargo from early endosomes to MVBs, and expression of a dominant negative VPS-4 mutant protein can significantly reduce trafficking from early endosomes to MVBs and the late endosome [52,53]. Neuronal expression of dominant negative VPS-4 from the transgene *nuIs145[P*_*glr-1*_::*vps-4(dn)]* blocks the movement of GLR-1 receptors from early endosomes to MVBs, and their subsequent turnover [54]. Expression of dominant negative VPS-4 in *rab-6*.*1* mutants restored GLR-1::GFP puncta number to wild-type levels (Fig 5A, 5B and 5C), indicating that the ESCRT pathway is required for GLR-1 turnover when retrograde transport via RAB-6.1 is blocked. Dominant negative VPS-4 also partially restored spontaneous reversal activity in *rab-6*.*1* mutants (Fig 5D), suggesting that some but not all GLR-1 receptors appear to be restored to their proper synaptic location in *rab-6*.*1; vps-4(dn)* animals.

If the pathways for recycling and turnover are both blocked, then GLR-1 should also accumulate in early endosomes. To investigate this possibility, we employed the endosomal marker SYX-7 (syntaxin 13), as this endosomal marker can be readily identified in the command neuron cell bodies [54]. We observed mRFP::SYX-7 and GLR-1::GFP colocalization in neuron cell bodies (Fig 5E, 5F and 5G) as previously described [21,51,54]. We did not observe a significant change in the amount of GLR-1::GFP colocalized with SYX-7::mRFP in *rab-6*.*1* mutants (Fig 5H), but we found a small but significant increase of colocalization in *rab-6*.*1; vps-4(dn)* mutants (from about 30% colocalization in *rab-6*.*1* mutants and in wild type to about 40% in *rab-6*.*1; vps-4(dn)* mutants). This result differs from our previous result from a similar analysis of *rab-6*.*2* mutants in which we found that nearly 70% of GLR-1::GFP colocalized with SYX-7::mRFP in *rab-6*.*2; vps-4(dn)* mutants, indicating that more of the stabilized GLR-1::GFP in *rab-6*.*2; vps-4(dn)* double mutants is trapped in endosomes than that in *rab-6*.*1; vps-4(dn)* double mutants. These colocalization results are consistent with our behavioral analysis of *rab-6*.*1* mutants, as *vps-4(dn)* can partially suppress behavioral defects in *rab-6*.*1* mutants but cannot suppress them in *rab-6*.*2* mutants [21].

### Constitutively active RAB-6.1 drives GLR-1 retrograde transport back to soma Golgi

**Fig 6:**
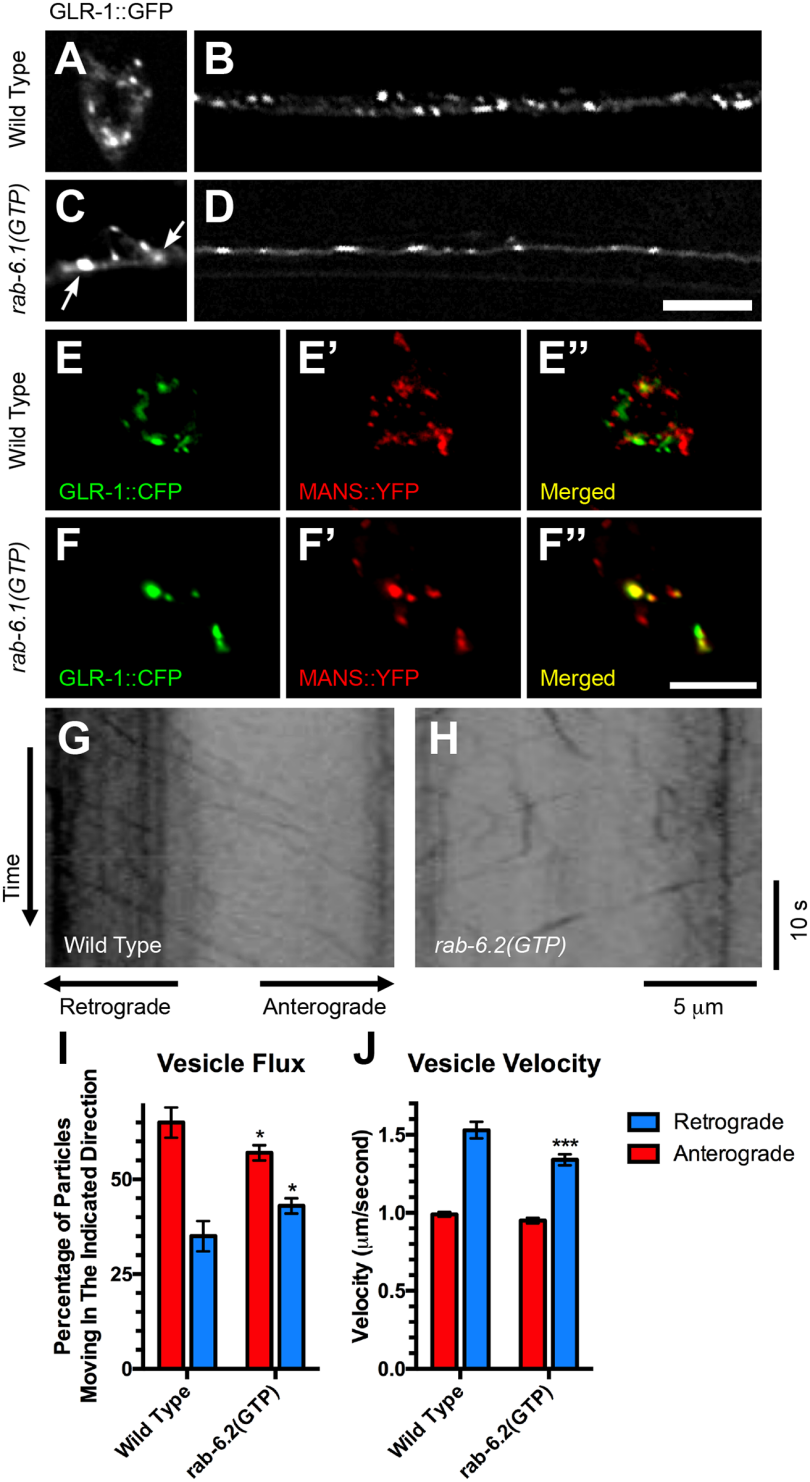
RAB-6.1 drives GLR-1::GFP from dendrites back to cell body Golgi. GLR-1::GFP in (A, B) wild type or (C, D) *rab-6*.*1(GTP)-*expressing transgenic animals. (E, F) GLR-1::CFP and (E’, F’) MANS::YFP fluorescence in cell bodies (PVC shown) for (E-E”) wild-type animals and (F-F”) animals expressing *rab-6*.*1(GTP)*. Merged images are shown in E” and F”. Bar, 5 μm. (G,H) Representative kymographs showing mobile and stationary GLR-1::GFP puncta in the anterior ventral cord of (G) wild-type animals or (H) animals expressing constitutively active RAB-6.2(GTP). For all kymographs, anterior (retrograde) is to the left. (I) Quantification of the fraction (percentage) of mobile GLR-1::GFP-containing vesicles moving in either a retrograde direction (blue bars) or an anterograde direction (red bars). (J) The mean velocity of GLR-1::GFP-containing vesicles as measured for anterograde and retrograde vesicles. Error bars are SEM. N = 285–362 vesicles. *p<0.01, Chi-square. ***P<0.001, Student t test.

Rab6 proteins can promote endosome to Golgi retrograde transport. We therefore tested whether RAB-6.1 activation is sufficient to promote retrograde transport of GLR-1. We generated a transgene expressing the GTPase defective RAB-6.1 (the constitutively active form) driven by the *glr-1* promoter and introduced the transgene into strains expressing GLR-1::GFP. We found that the expression of RAB-6.1(GTP) resulted in fewer GLR-1::GFP puncta in the ventral cord and an accumulation of GLR-1::GFP in several large puncta in the neuron cell bodies (Fig 6A–6D). To determine the subcellular location at which GLR-1 accumulates in the cell bodies of these animals, we performed colocalization studies between GLR-1::CFP and the Golgi marker mannosidase::YFP (MANS::YFP) [32,55], both expressed by the *glr-1* promoter. In wild-type cell bodies, little (34%±5%, n = 20) GLR-1::CFP is colocalized with MANS::YFP (Fig 6E–6E”), whereas most (83%±2%, n = 20, P<0.0001 compared to wild type via Student t-test) of the large GLR-1 puncta observed in animals that express RAB-6.1(GTP) was colocalized with MANS::YFP-decorated Golgi (Fig 6F–6F”). Thus, similar to activated RAB-6.2, activated RAB-6.1 is sufficient to promote the depletion of GLR-1 from dendrites and its accumulation at cell body Golgi.

Are the activated Rab6 proteins directly driving retrograde transport of GLR-1 from dendrites to cell bodies? Because RAB-6.2 has a larger impact on GLR-1 localization than does RAB-6.1, we decided to test whether activated RAB-6.2(GTP) can directly regulate the movement of GLR-1::GFP puncta along the ventral cord dendrites. We used time-lapse microscopy and kymograph analysis, photobleaching regions of the ventral cord just posterior to the AVG and RIG cell bodies to facilitate the visualization of GLR-1::GFP-containing vesicles moving over the bleached region as previously described [56–58]. In wild-type animals (Fig 6G), we observed that GLR-1::GFP puncta moved either in the anterior direction, towards the neuron cell bodies (i.e., retrograde) or in the posterior direction, away from the neuron cell bodies (i.e., anterograde). GLR-1::GFP-containing vesicles that moved in an anterograde direction had an average velocity of 1.0 μm/s, whereas retrograde vesicles had an average velocity of 1.5 μm/s (Fig 6J). Time-lapse analysis of GLR-1::GFP in animals expressing *rab-6*.*2(GTP)* revealed two differences in the movement of GLR-1::GFP vesicles compared with that in wild-type animals (Fig 6H, 6I and 6J). First, while the total number of moving vesicles was similar in both genotypes, with 0.70±0.10 (N = 285) moving vesicles per micron per minute for wild type versus 0.82±0.12 (N = 362) for *rab-6*.*2(GTP)*, 8% of the GLR-1::GFP-containing vesicles in flux switched from anterograde movement to retrograde movement in *rab-6*.*2(GTP)* animals compared to wild-type animals (Fig 6I; *p* < 0.01; Chi-square test). Second, the average velocity of vesicles moving retrograde in *rab-6*.*2(GTP)* animals was decreased by 12% relative to those in wild type (Fig 6J). Taken together with our previous findings [21] and the accumulation of GLR-1::GFP in the cell body of animals expressing *rab-6*.*1(GTP)* (Fig 6C), these results suggest that Rab6 proteins promote the retrograde transport of GLR-1::GFP from ventral cord dendrites back to cell body Golgi.

### RAB-6.1 is colocalized with RAB-6.2 at the Golgi in neuron cell bodies

**Fig 7:**
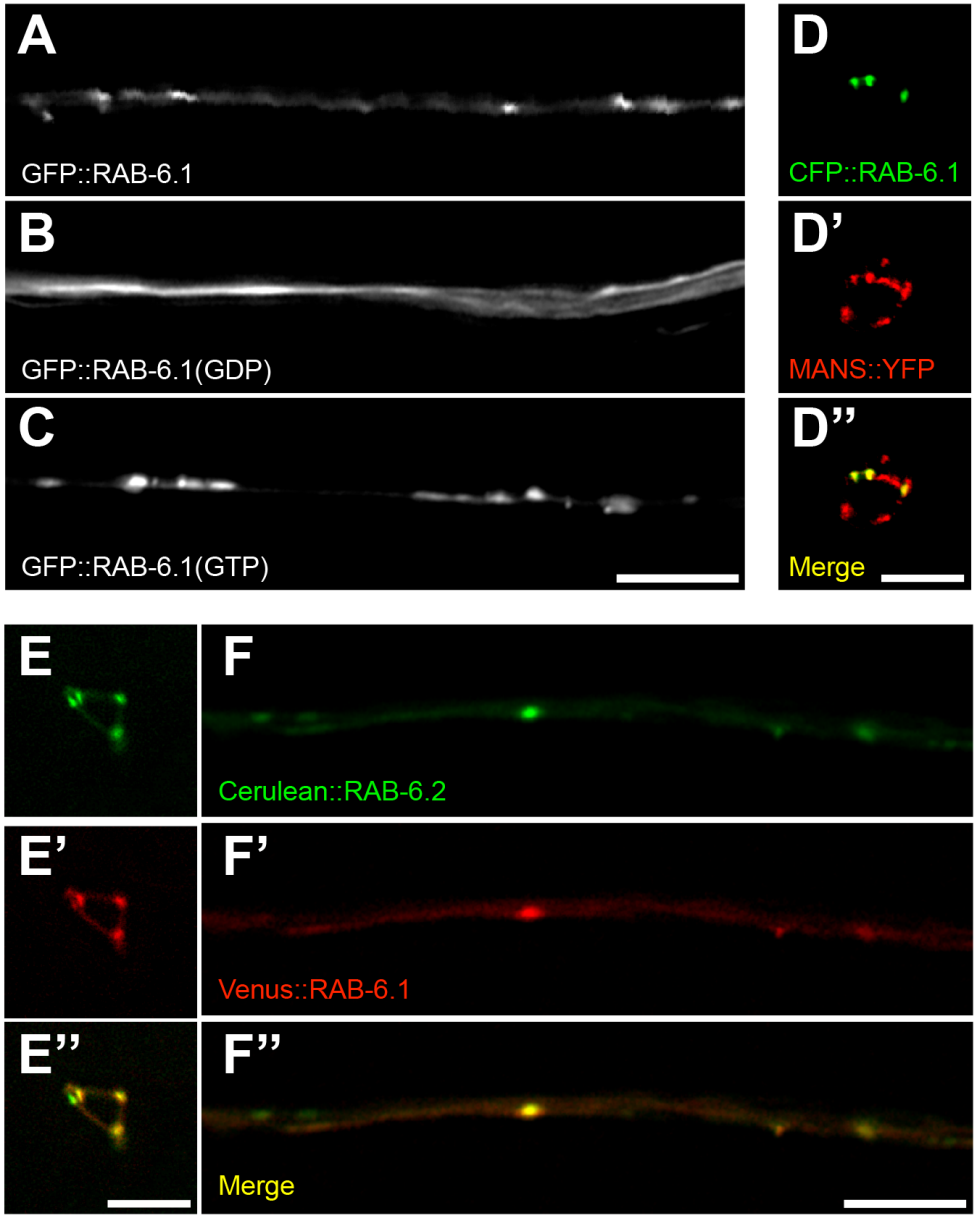
RAB-6.1 colocalizes with RAB-6.2 at the Golgi in neurons. (A,B,C) Subcellular localization of the indicated GFP::RAB-6.1 variant: (A) wild type, (B) a GDP-locked mutant, and (C) a GTP-locked mutant. (D) CFP::RAB-6.1 and (D’) MANS::YFP in interneuron cell bodies (PVC shown). (D”) Merged image showing CFP::RAB-6.1 colocalization to puncta with MANS::YFP. (E, F) Cerulean::RAB-6.2 and (E’, F’) Venus::RAB-6.1 in interneuron (E-E”) cell bodies (PVC shown) and (F-F”) ventral cord dendrites. Merged images (E”, F”) show colocalization at specific points. Bar, 5 μm.

To examine RAB-6.1 subcellular localization, we generated transgenes containing the *glr-1* promoter sequences fused to sequences encoding GFP fused in frame to the amino-terminal sequences of RAB-6.1. We found that RAB-6.1 is localized to punctate structures in along the ventral cord dendrites (Fig 7A). In addition to transgenic animals that expresses GFP::RAB-6.1, we also generated transgenic animals that express a CFP::RAB-6.1, finding that CFP::RAB-6.1 puncta colocalized with MANS::YFP-decorated Golgi in the neuron cell bodies (Fig 7D). We also examined the subcellular localization of the GDP-locked, dominant negative RAB-6.1 (Fig 7B) and the GTP-locked, constitutively active RAB-6.1 (Fig 7C). Dominant negative RAB-6.1(GDP) was diffusely localized, whereas RAB-6.1(GTP) was more tightly clustered into puncta than was wild-type RAB-6.1. Thus, activation of RAB-6.1 by GTP binding regulates its subcellular localization.

If RAB-6.1 and RAB-6.2 are functionally redundant, then they would be expected to act at the same subcellular sites within neurons. Like RAB-6.1, RAB-6.2 was shown previously to be colocalized with the Golgi marker MANS both in interneurons and intestinal epithelia [21]. We coexpressed Venus::RAB-6.1 and Cerulean::RAB-6.2 in the command interneurons and found that these two Rab6 isoforms are colocalized with each other in dendrites and cell bodies (Fig 7E–7E” and 7F–7F”). Consistent with their redundant function, these two GTPases are found in the same place within neurons.

### RAB-6.1 and RAB-6.2 regulate the recycling of retrograde cargo MIG-14

Given the synthetic lethal interaction of *rab-6*.*1* and *rab-6*.*2* mutations, combined with our finding that RAB-6.1 and RAB-6.2 redundantly promote the retrograde recycling of GLR-1 in neurons, we hypothesized that the two Rab6 proteins might also promote the recycling of well-characterized retrograde cargos in other tissues. Wntless homolog MIG-14 is one of the well-studied retromer cargos in *C*. *elegans*. Wntless is thought to act as a sorting receptor for the Wnt family of morphogens, shepherding them along the secretory pathway for eventual secretion into the extracellular space [36,38–43]. Retrograde transport of the Wntless/MIG-14 sorting receptor allows it to return to the Golgi to associate with a nascent Wnt cargo molecule for another round of secretion.

**Fig 8:**
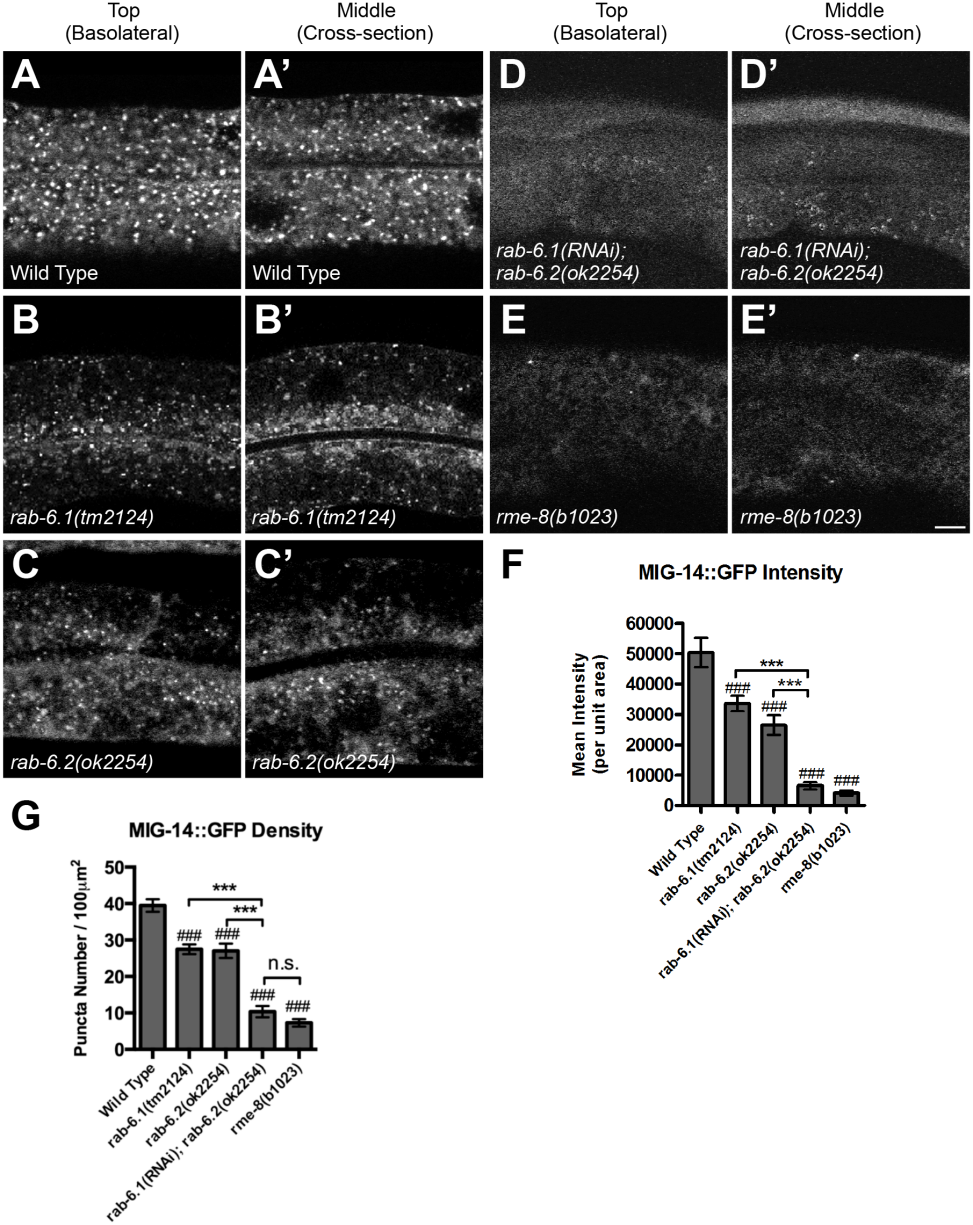
Recycling of MIG-14::GFP requires RAB-6.1 and RAB-6.2 activity. Intestinally expressed MIG-14::GFP in (A,B,C,D,E) top and (A’,B’,C’,D’,E’) middle focal planes of intestinal epithelial cells was examined by laser scanning confocal microscopy in (A) wild-type animals, (B) *rab-6*.*1(tm2124)* mutants, (C) *rab-6*.*2(ok2254)* mutants, (D) *rab-6*.*2(ok2254)* mutants treated by *rab-6*.*1* feeding RNAi since L1 stage, and (E) *rme-8(b1023)* mutants. (F,G) Quantified average MIG-14::GFP fluorescent intensity (F) and puncta number (G) for the indicated genotypes. Bar, 5 μm. Error bars are SEM. N = 24. ANOVA with Dunnett’s multiple comparison to wild type (###p<0.001) or Bonferroni Multiple Comparison test (***p<0.001).

**Fig 9:**
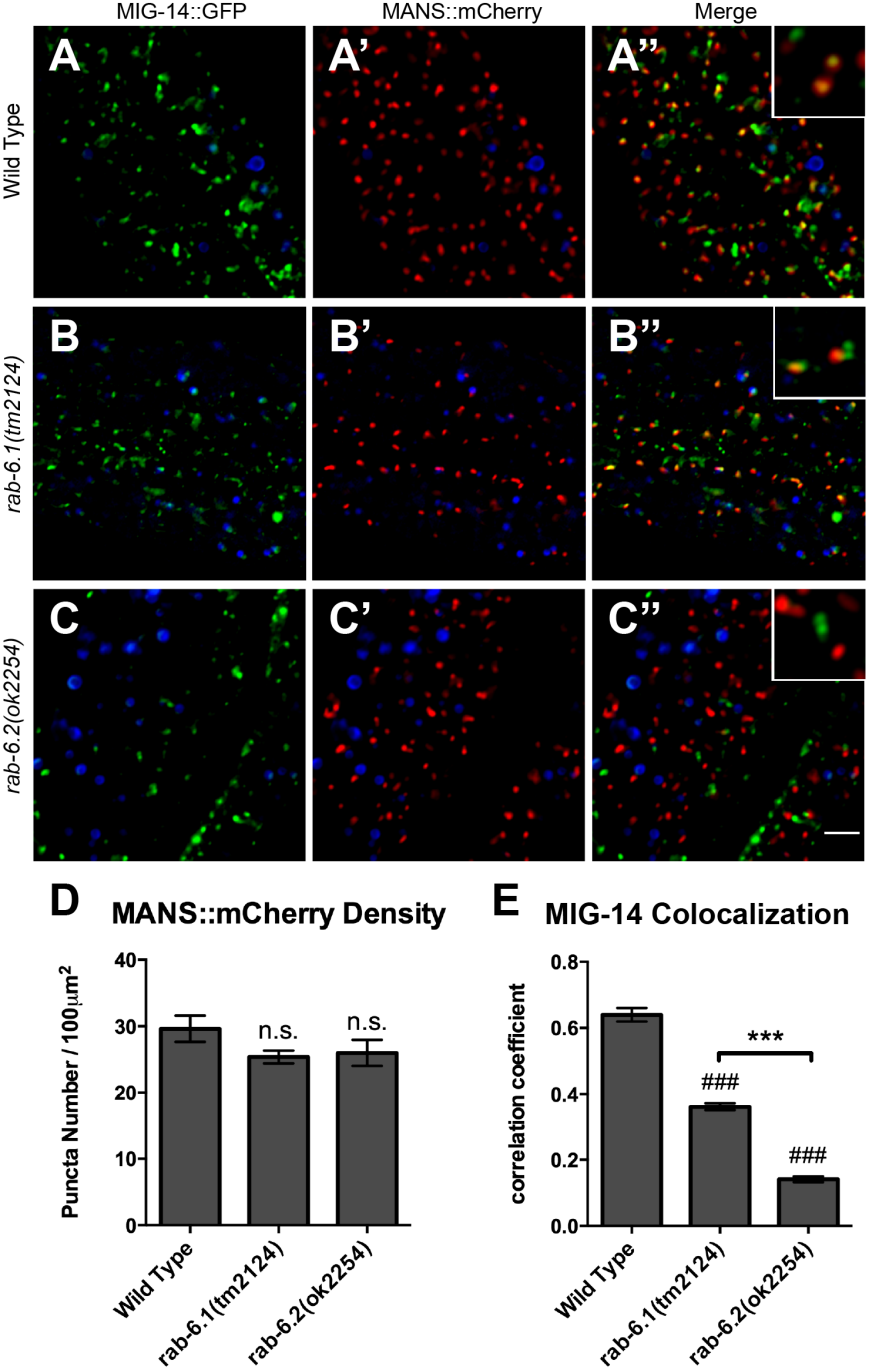
RAB-6.1 and RAB-6.2 direct MIG-14 back to Golgi. Fluorescent images were acquired from (A-A”) wild-type animals, (B-B”) *rab-6*.*1(tm2124)* mutants, or (C-C”) *rab-6*.*2(ok2254)* mutants expressing (A,B,C) MIG-14::GFP and (A’,B’,C’) MANS::mCherry. (A”,B”,C”) Merged images. Intestinal autofluorescent lysosome-like organelles are shown in the DAPI channel (blue). Bona fide fluorescence from GFP or RFP-tagged marker proteins was identified as fluorescence acquired in the green or red channels that also did not overlap with fluorescence acquired in the DAPI channel. MIG-14::GFP colocalized with Golgi marker MANS::mCherry in wild-type animals (A-A” and enlarged inset). Most MANS::mCherry-labeled Golgi ministacks lack MIG-14::GFP localization in *rab-6*.*1(tm2124)* mutants (B-B” and enlarged inset) and *rab-6*.*2(ok2254)* mutants (C-C” and enlarged inset), although MANS::mCherry and MIG-14::GFP puncta are typically adjacent (within 1 μm) in *rab-6*.*1* mutants. (D) Quantification of the number of MAN::mCherry puncta. (E) Quantification of MANS::mCherry and MIG-14::GFP colocalization as measured through average correlation coefficient. Bar, 5 μm. Error bars are SEM. N = 20. ANOVA with Dunnett’s multiple comparison to wild type (###p<0.001) or Bonferroni Multiple Comparison test (***p<0.001).

**Fig 10:**
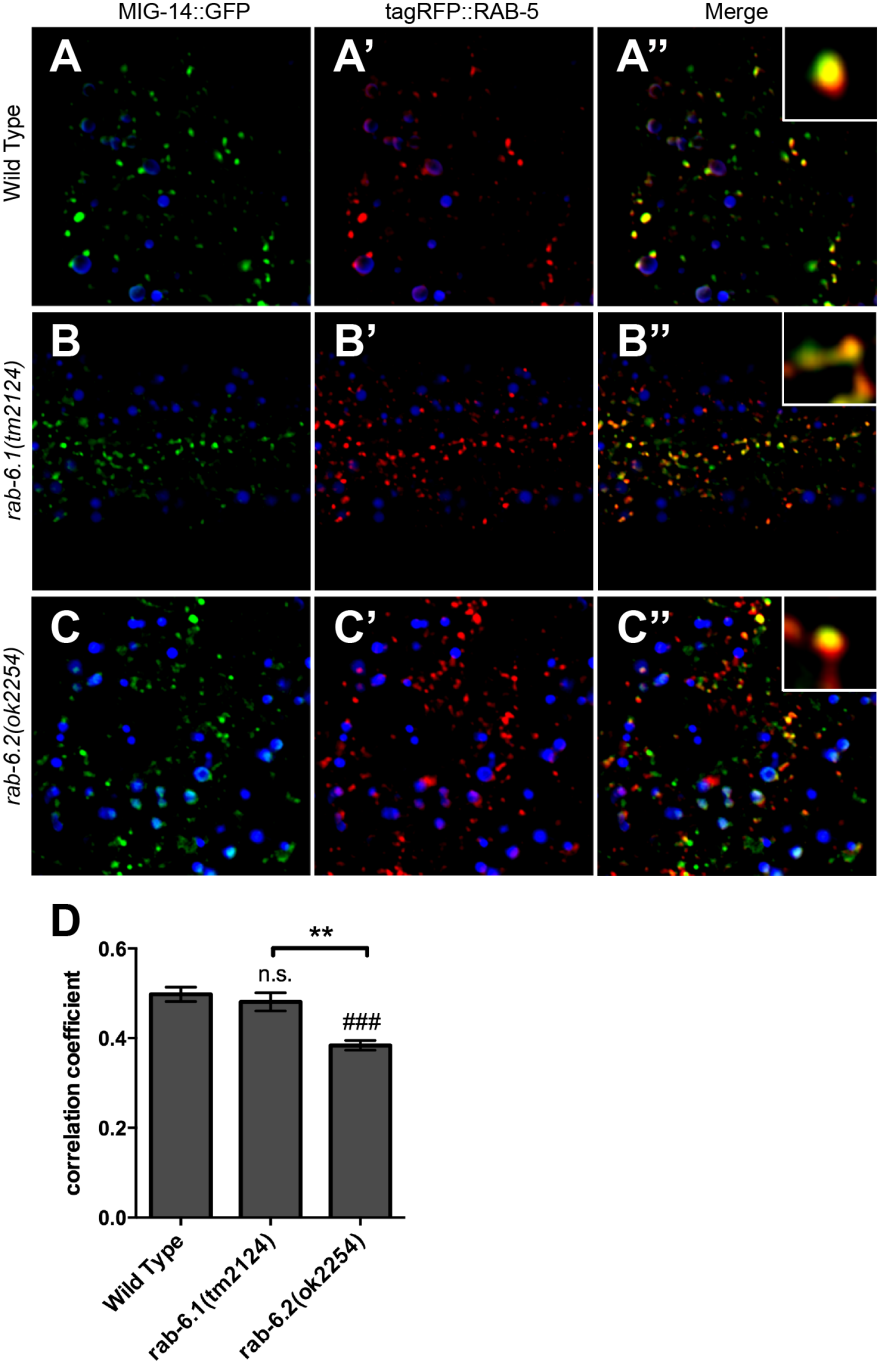
RAB-6.1 and RAB-6.2 drive MIG-14 out of early endosomes. Fluorescent images were acquired in (A-A”) wild-type animals, (B-B”) *rab-6*.*1(tm2124)* mutants, or (C-C”) *rab-6*.*2(ok2254)* mutants expressing (A,B,C) MIG-14::GFP and (A’,B’,C’) tagRFP::RAB-5. Intestinal autofluorescent lysosome-like organelles are shown in the DAPI channel (blue). MIG-14::GFP colocalized with tagRFP::RAB-5 labeled early endosomes in wild-type animals (A-A” and enlarged inset), *rab-6*.*1(tm2124)* mutants (B-B” and enlarged inset), and *rab-6*.*2(ok2254)* mutants (C-C” and enlarged inset). (D) Quantification of tagRFP::RAB-5 and MIG-14::GFP colocalization as measured through average correlation coefficient. Bar, 5 μm. Error bars are SEM. N = 20. ANOVA with Dunnett’s multiple comparison to wild type (###p<0.001) or Bonferroni Multiple Comparison test (**p<0.01).

A model for the retrograde recycling of MIG-14 has been well established in the *C*. *elegans* intestine using a transgene that expresses a MIG-14::GFP chimeric protein from the *vha-6* intestinal promoter [36]. In wild type intestinal cells, MIG-14::GFP accumulates in punctate structures (Fig 8A), and these puncta are colocalized with either Golgi (Fig 9A) or early endosomal markers (Fig 10A) [36]. In mutants that are defective in retrograde recycling, including those for the retromer genes *snx-1* and *vps-35*, as well as the retromer-associated gene *rme-8*, MIG-14::GFP is shunted to late endosomes and lysosomes, resulting in a significant drop in the fluorescent intensity of MIG-14::GFP puncta [36]. We introduced the *P*_*vha-6*_::*mig-14*::*gfp* transgene into both *rab-6*.*1* and *rab-6*.*2* mutants, where we observed a quantifiable decrease in MIG-14::GFP fluorescence and puncta number for both mutants (Fig 8B, 8C, 8F and 8G), although not to the same extent as that observed in *rme-8* mutants (Fig 8E, 8F and 8G). Our results indicate that both Rab6 proteins have a more general role in retrograde recycling.

We next examined MIG-14::GFP in animals lacking both RAB-6.1 and RAB-6.2 activity. The *rab-6*.*1* and *rab-6*.*2* mutations are synthetic lethal, excluding the possibility of directly introducing the *P*_*vha-6*_::*mig-14*::*gfp* transgene into *rab-6*.*1; rab-6*.*2* double mutants. However, *C*. *elegans* intestinal cells are vulnerable to RNAi by feeding, allowing us to examine animals impaired for both genes by performing feeding RNAi for *rab-6*.*1* in a *rab-6*.*2* knock out background, i.e. *rab-6*.*1(RNAi); rab-6*.*2(ok2254)*. We grew *rab-6*.*2* mutant L1-stage animals on *rab-6*.*1(RNAi)* media and examined the resulting L4-stage larvae. We found that the intensity and number of MIG-14::GFP puncta in *rab-6*.*1(RNAi); rab-6*.*2(ok2254)* mutants dropped to the low level observed in *rme-8* mutants (Fig 8D–8G), providing additional support for a redundant role for these Rabs in retrograde recycling.

**Fig 11:**
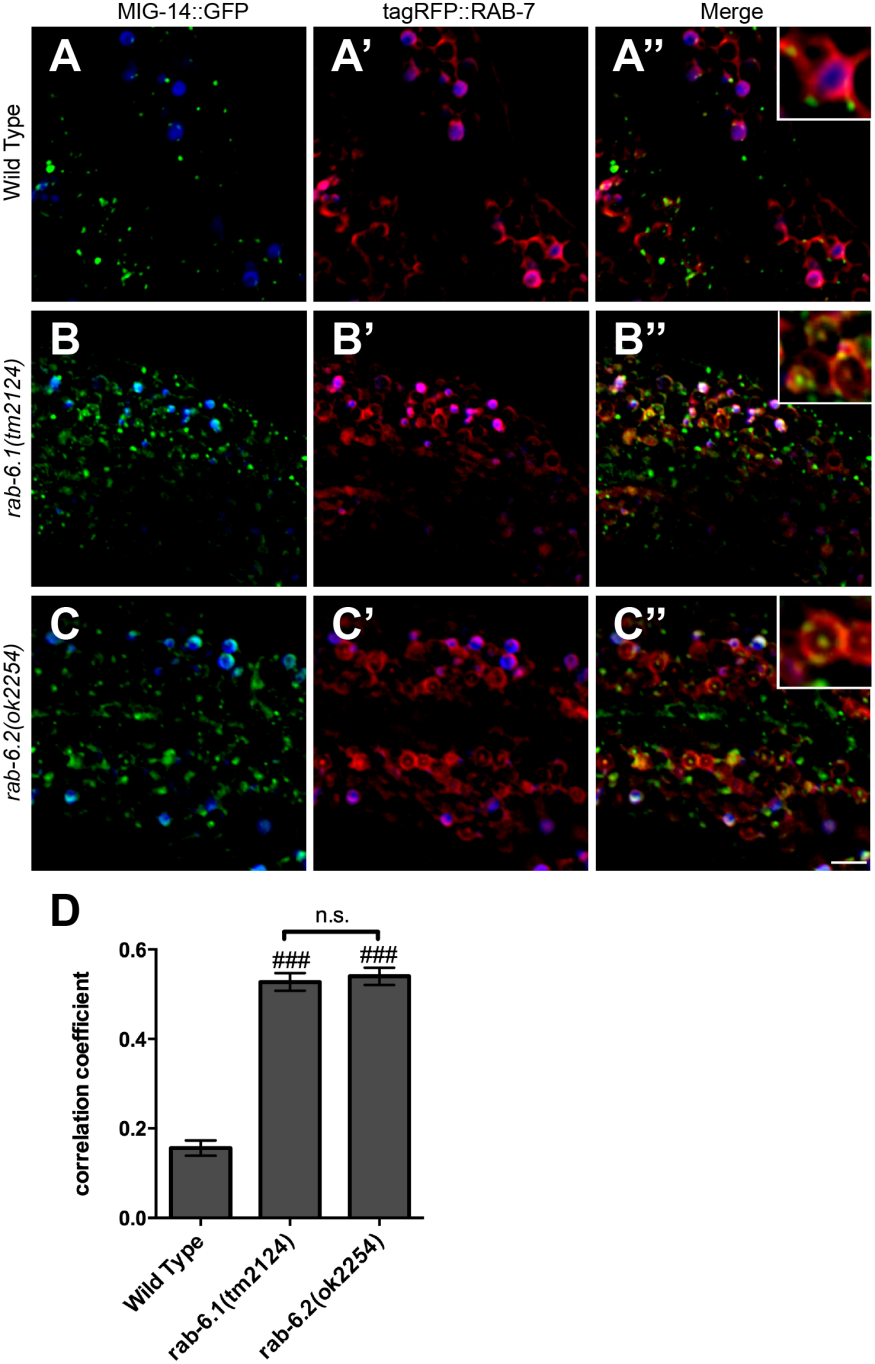
RAB-6.1 and RAB-6.2 drive MIG-14 away from late endosomes. Fluorescent images were acquired in (A-A”) wild-type animals, (B-B”) *rab-6*.*1(tm2124)* mutants, or (C-C”) *rab-6*.*2(ok2254)* mutants expressing (A,B,C) MIG-14::GFP and (A’,B’,C’) tagRFP::RAB-7. Intestinal autofluorescent lysosome-like organelles are shown in the DAPI channel (blue). MIG-14::GFP localized to the apparent limiting membrane and intralumenal structures of late endosomes/lysosomes (most likely MVBs) labeled by tagRFP::RAB-7 in *rab-6*.*1(tm2124)* mutants (B-B” and enlarged inset) and *rab-6*.*2(ok2254)* mutants (C-C” and enlarged inset), but not in wild-type animals (A-A” and enlarged inset). (D) Quantification of tagRFP::RAB-5 and MIG-14::GFP colocalization as measured through average correlation coefficient. Bar, 5 μm. Error bars are SEM. N = 20. ANOVA with Dunnett’s multiple comparison to wild type (###p<0.001).

In the absence of retrograde recycling, MIG-14 cannot return to Golgi, but instead ends up in both early and late endosomes [36]. We directly tested whether MIG-14 returns to intestinal Golgi in the absence of the Rab6 proteins by introducing the *P*_*vha-6*_::*mig-14*::*gfp* transgene into *rab-6*.*1* or *rab-6*.*2* mutants that also express either the Golgi marker mannosidase::mCherry (MANS::mCherry), the early endosome marker tagRFP::RAB-5, or the late endosome marker tagRFP::RAB-7 [36]. Whereas MIG-14::GFP and MANS::mCherry are colocalized in wild-type animals (Fig 9A, 9D and 9E), we found that there is little colocalization between MIG-14::GFP and MANS::mCherry in *rab-6*.*1* mutants or *rab-6*.*2* mutants (Fig 9B–9E). Instead, we found that in *rab-6*.*1* mutants or *rab-6*.*2* mutants, MIG-14::GFP accumulates either in early endosomes (Fig 10B, 10C and 10D) or in ring-like late endosomes and lysosomes (Fig 11B, 11C and 11D). These defects in MIG-14::GFP localization are similar to those observed in known retromer mutants [36]. Interestingly, in *rab-6*.*1* mutants, MIG-14::GFP accumulates in endosomes that are adjacent to Golgi, whereas there is no such spatial coordination in *rab-6*.*2* mutants (Fig 9B” and 9C”), indicating that the two Rab6 proteins do have distinct roles, at least in the intestine. Taken together, our findings indicate that RAB-6.1 and RAB-6.2 promote the membrane recycling of retromer cargos to the Golgi, and in the absence of RAB-6.1 or RAB-6.2 activity, such cargo accumulates in distinct sorting endosomes before being shunted to late endosomes and lysosomes for turnover.

### RAB-6.1 is localized to intestinal Golgi

**Fig 12:**
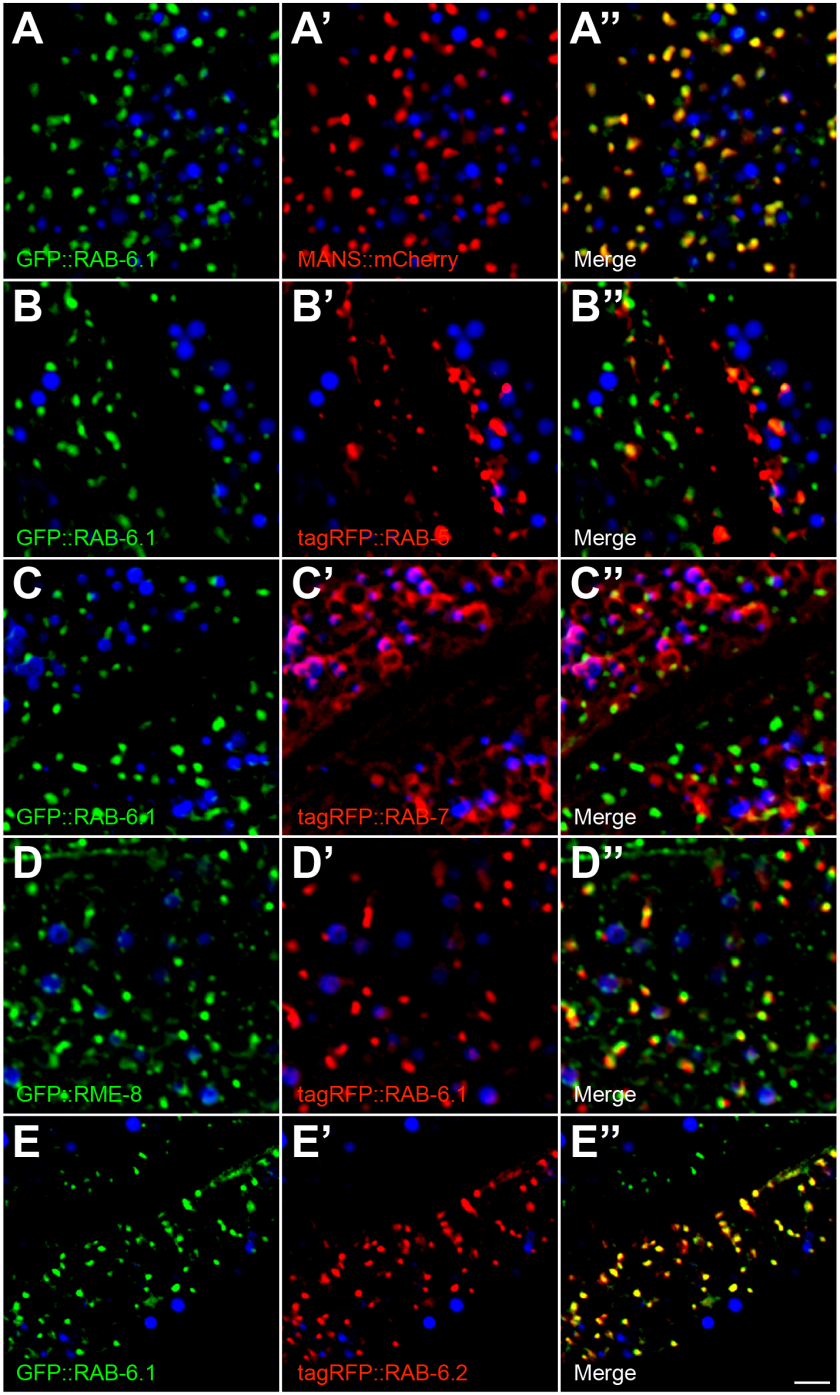
RAB-6.1 colocalizes with Mannosidase/MANS, RME-8 and RAB-6.2, but not with RAB-5 and RAB-7 in intestinal epithelial cells. GFP-, tagRFP-, or mCherry-tagged proteins were expressed in intestinal epithelial cells specifically using the *vha-6* promoter. Fluorescent images are shown for the indicated markers in wild-type animals. (A) GFP::RAB-6.1 is colocalized with (A’) Golgi marker MANS::mCherry. (B,C) GFP::RAB-6.1 is not colocalized with (B’) early endosome marker tagRFP::RAB-5 and (C’) late endosome/lysosome marker tagRFP::RAB-7. (D) GFP::RME-8 is localized near (D’) tagRFP::RAB-6.1. GFP::RME-8 and tagRFP::RAB-6.1 do not overlap, but are adjacent (within 1 μm). (E) GFP::RAB-6.1 is colocalized with (E’) tagRFP::RAB-6.2. (A”-E”) Merged images are shown. Intestinal autofluorescent lysosome-like organelles are shown in the DAPI channel (blue) in all panels. Bar, 5 μm.

Is RAB-6.1 recruited to Golgi in intestinal cells, similar to its subcellular localization in neurons? To test this possibility through colocalization analysis, we placed sequences encoding a GFP::RAB-6.1 chimera under the control of the intestine-specific *vha-6* promoter and introduced the resulting transgene into animals that either expressed MANS::mCherry, tagRFP::RAB-5, tagRFP::RAB-7, or tagRFP::RAB-6.2 in their intestines [21]. In addition, we also generated a *P*_*vha-6*_::*tagrfp*::*rab-6*.*1* transgene and examined colocalization between tagRFP::RAB-6.1 and GFP::RME-8 [59]. We found that GFP::RAB-6.1 and MANS::mCherry are precisely colocalized with each other (Fig 12A, Table 1), indicating that RAB-6.1 resides at Golgi in intestine as well as in neurons (Fig 7D–7F). By contrast, little GFP::RAB-6.1 was colocalized with tagRFP::RAB-5 or tagRFP::RAB-7 (Fig 12B and 12C, Table 1). We also examined colocalization with RME-8, a J domain protein that associates with the retromer complex, where it helps regulate clathrin dynamics at early endosomes [36]. RME-8 is colocalized with a subgroup of RAB-5-containing structures that are found adjacent to the Golgi [36]. As predicted from its precise colocalization with the Golgi marker MANS::mCherry, tagRFP::RAB-6.1 puncta were found adjacent to puncta containing GFP::RME-8 (Fig 12D, Table 1). We also examined the colocalization of GFP::RAB-6.1 and tagRFP::RAB-6.2, finding precise colocalization of these two proteins to the same punctate structures in the intestine (Fig 12E, Table 1). This is consistent with our previous finding that RAB-6.2 was localized to puncta that were adjacent to RME-8-containing puncta [21]. Our findings suggest that both Rab6 proteins accumulate primarily at the Golgi, and that if they are localized to distinct structures, those structures are so close together as to be below the level of detection for light microscopy. Taken together, our findings in *C*. *elegans* neuronal and intestinal cells suggest that RAB-6.1, RAB-6.2, and RME-8, along with the retromer complex, are localized along an endosome-Golgi axis, where they together promote the retrograde recycling of cargo molecules like GLR-1 and MIG-14.

**Table 1:** Colocalization in the Intestine.

Protein 1	Protein 2	Correlation Coefficient	N
GFP::RAB-6.1	MANS::mCherry	0.81±0.05	20
GFP::RAB-6.1	tagRFP::RAB-5	0.25±0.03	20
GFP::RAB-6.1	tagRFP::RAB-7	0.25±0.04	20
GFP::RME-8	tagRFP::RAB-6.1	0.69±0.03	20
GFP::RAB-6.1	tagRFP::RAB-6.2	0.78±0.04	20

Colocalization (as measured as a correlation coefficient) of the indicated proteins in each row when co-expressed in the intestine.

## Discussion

We have shown that like RAB-6.2, the small GTPase RAB-6.1 also regulates retrograde trafficking of the AMPAR subunit GLR-1 in *C*. *elegans*. Levels of GLR-1 are decreased in *rab-6*.*1* mutants due to turnover, although not to the same degree as observed in *rab-6*.*2* mutants. Animals lacking *rab-6*.*1* gene function also show GLR-1-related behavioral defects. In addition, constitutively active RAB-6.1 drives GLR-1 back to Golgi in the cell body. Loss of both Rab6 proteins causes a more dramatic phenotype than loss of either single Rab6 alone, indicating that these two proteins redundantly control GLR-1 trafficking. In addition, we used a well-studied retrograde transport cargo, MIG-14, to investigate the functions of RAB-6.1 and RAB-6.2 in general retrograde trafficking. In *rab-6*.*1* mutants, MIG-14 accumulates in early endosomes that are adjacent to Golgi (and decorated with RME-8), whereas in *rab-6*.*2* mutants, MIG-14 accumulates in early endosomes that are located distal from Golgi, suggesting that these proteins might regulate different steps of retrograde recycling or spatially distinct endosomal compartments.

Are RAB-6.1 and RAB-6.2 acting redundantly and conducting the same molecular and cellular functions, or do they have distinct and dedicated functions? Mutations for both genes show a synthetic genetic interaction when placed into the same animal, indicating that they have some redundancy. Yet both genes have clear phenotypes as single mutants, indicating that there are some aspects of function that are non-redundant. Both proteins appear to be expressed in nearly all tissues, suggesting that differences in expression pattern are not likely to explain why single mutants in either gene result in clear (non-redundant) phenotypes. Thus, the answer to this question of redundancy might depend on the specific cargo being recycled and the specific tissue and cell type being examined.

**Fig 13:**
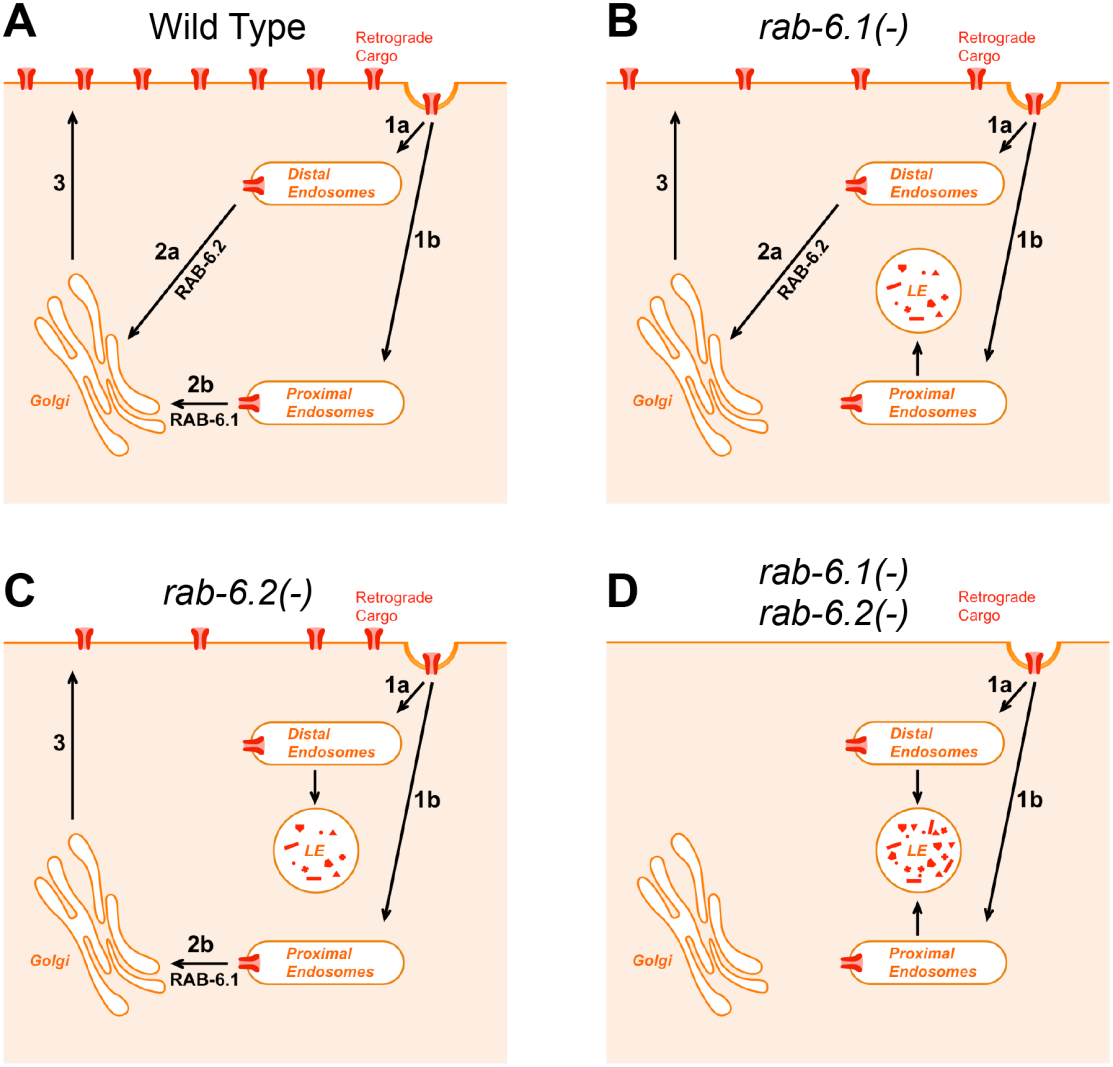
A model for RAB-6.1 and RAB-6.2 function. (A) In wild-type animals, retrograde cargo undergoes endocytosis from the plasma membrane (arrows 1a and 1b). Endocytosed cargo is delivered to early endosomes that are either (1a) distal from or (1b) proximal to Golgi. Cargo then undergoes retrograde recycling driven either by RAB-6.2 (arrow 2a) if at Golgi-distal endosomes or by RAB-6.1 (arrow 2b) if at Golgi-proximal endosomes. (3) After delivery to Golgi, recycled cargo is redelivered to the plasma membrane. (B,C) In either (B) *rab-6*.*1* mutants or (C) *rab-6*.*2* mutants, recycling through Golgi-proximal endosomes is blocked, resulting in some cargo being delivered to lysosomes for degradation. (D) In animals lacking both Rab6 proteins, recycling of retrograde cargo is entirely blocked, resulting in complete degradation.

The trafficking of MIG-14 in intestinal cells is informative, as it accumulates in distinct (spatially segregated) subgroups of early endosomes in *rab-6*.*1* mutants compared to *rab-6*.*2* mutants (Figs 9 and 10). Specifically, when we performed colocalization studies between MIG-14::GFP and MANS::mCherry in *rab-6*.*1* and *rab-6*.*2* mutants, we found that the colocalization patterns are slightly different. On one hand, although MIG-14::GFP and MANS::mCherry are not precisely colocalized, they nevertheless show an adjacent colocalization pattern in *rab-6*.*1* mutants (Fig 9B–9B”). On the other hand, MIG-14::GFP and MANS::mCherry are localized away from each other in totally separated structures in *rab-6*.*2* mutants (Fig 9C–9C”). These results could imply that RAB-6.1 and RAB-6.2 regulate retrograde recycling from two types of early endosomes–Golgi-proximal and Golgi-distal–with RAB-6.1 mediating the exit out of Golgi-proximal endosomes and RAB-6.2 mediating the exit out of Golgi-distal endosomes (Fig 13A). Typical retrograde cargo would move through both endosome types and thus be dependent on both Rab6 genes. Consistent with this idea of separate endosome types, retromer-related protein RME-8 decorates endosomes that are adjacent to Golgi. In this model, loss of either Rab6 gene alone results in failed recycling of some but not all cargo like MIG-14 (Fig 13B and 13C), whereas loss of both Rab6 proteins completely shuts down all recycling pathways (Fig 13D), which is consistent with our findings.

Alternatively, RAB-6.1 and RAB-6.2 might act sequentially in the same pathway, with RAB-6.2 promoting exit of cargo from early endosomes and RAB-6.1 promoting docking of those cargo vesicles at the Golgi. RAB-6.2 might help to accumulate retrograde cargoes in specialized early endosomes and then the retromer and RAB-6.1 carry out the final step of retrograde trafficking from these special endosomes to the Golgi. The differences in the molecular function of these two Rab6 proteins might be in the specific effectors that they each recruit. RAB-6.2 might recruit motor proteins to help long-distance trafficking of the cargo from distal endosomes to proximal endosomes, whereas RAB-6.1 might recruit tethering, docking, and fusion factors that mediate the final fusion step at the Golgi. While this model does not explain why loss of both Rab6 proteins results in an additive phenotype, it is possible that each Rab6 can fill in for the other, at least partly, if the other is impaired.

Even considering the regulation of GLR-1 trafficking in neurons, the functions of RAB-6.1 and RAB-6.2 are not likely to be identical. First, the defects observed in GLR-1 trafficking in *rab-6*.*1* mutants and *rab-6*.*2* mutants are not identical. Specifically, *rab-6*.*1* mutants always have a more modest phenotype than *rab-6*.*2* mutants (Fig 2), with the caveat that RAB-6.1 is expressed in all but one of the command interneurons (AVA) whereas RAB-6.2 is expressed in all of the command interneurons. Second, the subcellular localization of GLR-1 is not the same in *rab-6*.*1* mutants compared to *rab-6*.*2* mutants when blocking degradation of GLR-1. The percentage of GLR-1 colocalized with the early endosome marker SYX-7 is nearly 70% in *rab-6*.*2* mutants [21], whereas that number is only a little larger than 40% in *rab-6*.*1* mutants (Fig 5H). These colocalization results are consistent with our behavioral analysis of these mutants, in which *vps-4(dn)* can partially suppress *rab-6*.*1* mutants but not *rab-6*.*2* mutants (Fig 5D and [21]). Our behavior and colocalization results suggest that only a small amount of GLR-1 accumulates in early endosomes in *rab-6*.*1* mutants when GLR-1 degradation is blocked, whereas the remainder is properly localized, most likely through recycling by a RAB-6.2-dependent process. Taken together, our results suggest that RAB-6.2 is probably the primary regulator of GLR-1 retrograde trafficking, and RAB-6.1 might play a complementary role as a secondary regulator for GLR-1 transport regulation. It remains possible that the weight of their respective roles changes depending on environment and/or prior experience.

While the two Rab6 proteins shown partial functional redundancy for GLR-1 and MIG-14 trafficking, they appear to have non-redundant roles in fertility. We found that *rab-6*.*2* null mutants are viable and fertile, whereas *rab-6*.*1* null mutants are sterile. Interestingly, we found that *rab-6*.*1* mutants are capable of developing to the adult stage normally without any delays or visible morphological defects, but lay unfertilized oocytes instead of normal fertilized eggs. This fertilization defect can be rescued by mating to wild-type males. Therefore, RAB-6.1, but not RAB-6.2, is required for either proper sperm function or spermatogenesis in *C*. *elegans*.

Multiple isoforms are common for the Rab GTPase family. While yeast has only the Rab6 protein Ypt6, there are four mammalian Rab6 isoforms (Rab6A, A’, B and C), each with distinct functions. Rab6A and Rab6A’ are splicing alternatives encoded by the same gene, Rab6B is encoded by a separate gene, and Rab6C is retrogene derived from a Rab6A' transcript during primate evolution. It is likely that the Rab6 founding gene diverged into two genes (Rab6A and Rab6B) during early metazoan evolution. Consistent with this hypothesis, *C*. *elegans* has two Rab6 proteins, and we find that RAB-6.1 is the likely ortholog of Rab6A and RAB-6.2 is the likely ortholog of Rab6B. Rab6B is expressed in the nervous system [17], and RAB-6.2 has the more critical role in GLR-1 recycling in neurons, suggesting that the Rab6B/RAB-6.2 subfamily might have become specialized for neurons early in evolution. We speculate that this subfamily recruits effectors that are tailored for neuron cell biology. The identity of the specific effectors employed by these two Rab6 subfamilies, particularly with regard to different cell types, will be the next big challenge.

## Materials and Methods

### Strains

Animals were grown at 20°C on standard NGM plates seeded with OP50 *E*. *coli*. Some strains were provided by the *Caenorhabditis* Genetics Center. Strains were backcrossed to our laboratory N2 strain to minimize other genetic variation. The mutants *rab-6*.*1(tm2032)* and *rab-6*.*1(tm2124)*, which could not be initially maintained as homozygotes, were crossed to the balancer *hT2[bli-4(e937) let-*?*(q782) qIs48](I*, *III)* to generate balanced strains. In addition, the following strains were used: *glr-1(ky176)*, *nuIs108[P*_*glr-1*_::*glr-1(4kr)*::*gfp]*, *nuIs145[P*_*glr-1*_::*vps-4(dn)]* (a gift from J. Kaplan, Mass. General Hospital, Boston, MA), *nuIs25[P*_*glr-1*_::*glr-1*::*gfp]*, *odEx[P*_*glr-1*_::*mans*::*yfp]*, *odEx[P*_*glr-1*_::*venus*::*rab-6*.*1(+)]*, *odEx[P*_*glr-1*_::*cerulean*::*rab-6*.*2(+)]*, *odEx[P*_*glr-1*_::*gfp*::*rab-6*.*1(+)]*, *odEx[P*_*glr-1*_::*gfp*::*rab-6*.*1(gdp)]*, *odEx[P*_*glr-1*_::*gfp*::*rab-6*.*1(gtp)]*, *odEx[P*_*glr-1*_::*mrfp*::*syx-7]*, *odEx[P*_*vha-6*_::*gfp*::*rab-6*.*1(+)]*, *pwEx102[P*_*vha-6*_::*mans*::*mcherry]*, *odEx[P*_*vha-6*_::*tagrfp*::*rab-6*.*2(+)]*, *odIs1[P*_*glr-1*_::*snb-1*::*gfp]*, *pwIs798[P*_*vha-6*_::*mcherry*::*rme-8]*, *pwIs825[P*_*vha-6*_::*gfp*::*rme-8]*, *pwIs846[P*_*vha-6*_::*tagrfp*::*rab-5]*, *pwIs849[P*_*vha-6*_::*tagrfp*::*rab-7]*, and *rab-6*.*2(ok2254)*.

### Transgenes and germline transformation

Transgenic strains generated in this study were isolated after microinjecting various plasmids (5–50 ng/ml) using *rol-6dm* (a gift from C. Mello, University of Mass. Medical School, Worcester, MA), *ttx-3*::*rfp*, or *ttx-3*::*gfp* (a gift from O. Hobert, Columbia Univ. College of Physicians and Surgeons, New York, NY) as a marker. Plasmids containing the *glr-1* promoter (a gift from V. Maricq, Univ. of Utah, Salt Lake City, UT) or *vha-6* promoters (a gift from B. Grant, Rutgers University), followed by the indicated cDNA construct, were generated using standard techniques. Plasmids for examining *rab-6*.*1* gene expression were made by PCR amplification of 3 kb of upstream promoter sequence as well as the complete coding sequences and introns for the *rab-6*.*1* transcription unit. The resulting PCR product was then subcloned into a GFP expression vector. All resulting neuronal transgenes were introduced into the germline and followed as extrachromosomal arrays. All intestinal transgenes were obtained using low-copy particle bombardment. The fluorophore-tagged reporters described in this manuscript have been previously demonstrated to be functional, except for RAB-5, RAB-7, and mannosidase, as mutants for their corresponding genes either do not exist or are lethal and thus cannot be easily tested for functionality.

### Fluorescence microscopy

GFP- and RFP-tagged fluorescent proteins were visualized in nematodes by mounting young adult animals on 2% agarose pads with levamisole. Fluorescent images were observed using a Zeiss Axioplan II. A 100X (N.A. = 1.4) PlanApo oil immersion objective was used to detect GFP, Venus, Cerulean, mCherry, CFP, YFP, and mRFP signals of worms mounted on 2% agarose pads at room temperature. Imaging was done with an ORCA ERG 1394 charge-coupled device (CCD) camera (Hamamatsu, Bridgewater, NJ) using iVision v4.0.11 (Biovision Technologies, Exton, PA) software. Exposure times were chosen to fill the 12-bit dynamic range without saturation. Maximum intensity projections of z-series stacks were obtained and out-of-focus light was removed with a constrained iterative deconvolution algorithm (iVision by Biovision Technologies, Exton, PA). For most images, we captured the ventral cord dendrites in the retrovesicular ganglion region posterior to the RIG and AVG cell bodies.

The quantification of ventral nerve cord fluorescent objects (i.e., puncta and enlarged endosomes) was done using ImageJ to automatically threshold the images and then determine the outlines of fluorescent objects in ventral cord dendrites. ImageJ was used to quantify the number of individual fluorescent puncta along the ventral cord (counted and normalized per 100 microns of length).

The quantification of PVC cell body fluorescence was done using ImageJ to measure the integrated fluorescent density (the sum of all detectable pixel intensities per cell body) for each neuron. For the quantification of GLR-1::GFP and mRFP::SYX-7 colocalization, we fixed animals with ice cold 1% paraformaldehyde in PBS for 10 minutes and imaged them using a previously published protocol [51,54]. Images for neuronal cell bodies were taken using a Carl Zeiss confocal microscope equipped with the BD CARV IITM Confocal Imager and a Carl Zeiss 100X Plan-Apochroma objective (N.A. = 1.4).

For quantitative colocalization analysis of GLR-1, all image manipulations were performed with iVision v4.0.11 (Biovision Technologies, Exton, PA) software using the FCV colocalization function. We applied an empirically derived threshold to all images for both the GLR-1::GFP channel and the mRFP::SYX-7 channel to eliminate background coverslip fluorescence and other noise (typically, <5% of pixels for each channel). The fluorescent intensity values for both the GLR-1::GFP and mRFP::SYX-7 channels were then graphed on a scatter plot to analyze individual pixels. Pixels with similar intensity values for both channels (within an empirically established tolerance factor) were counted as colocalized. To acquire the fraction of GLR-1::GFP colocalized with mRFP::SYX-7, the number of colocalized pixels was normalized to the number of GLR-1::GFP pixels under threshold. To maximize our resolving power while observing the relatively small *C*. *elegans* neuron cell bodies, we restricted our analysis to a single focal plane taken through the middle of each cell body.

For quantification of intestinal puncta colocalization, all images were analyzed in ImageJ using the plugin Coloc2 to calculate a Pearson’s correlation value to images that were under threshold as described above.

### Time-lapse imaging and data analysis

For all in vivo time-lapse imaging of the ventral nerve cord, 1-day old adult animals with the *nuIs25[glr-1*::*gfp]* transgene were immobilized with 3 mM levamisole in M9 and mounted on a 2% agarose pad. Imaging was performed in an anterior region (70–80 μm) of the ventral cord just posterior to the RIG and AVG neuronal cell bodies. GLR-1::GFP time-lapse images were obtained with an Olympus IX81 microscope (Olympus, Tokyo, Japan) using a Plan Apochromat objective (100X, 1.4 NA) attached with a spinning disk confocal head (CSU22; Yokogawa, Tokyo, Japan) and equipped with an electron-multiplying charge-coupled device (EMCCD) camera (iXon 897; Andor Technology, South Windsor, CT). A 488 nm laser (25 mW) was used at 10% power for imaging (285 ms exposure time). This same laser was used at 20% power to photobleach the ventral cord, which allowed for improved confidence in visualizing moving vesicles in kymographs. We focused on the VNC using the 488-nm laser at 10% power (100X objective, 285-ms exposure). Moving particles were defined as puncta that were displaced by at least three pixels in successive time frames, and stationary particles were defined as puncta that were immobile for more than three consecutive frames. The flux of particles was calculated as the number of puncta moving in either direction in a 15- to 20-μm region (just posterior to the RIG and AVG cell bodies) divided by the total time. Run length was calculated as the length in microns of each distinct movement event in the *x*-coordinate plane. The start and end of each movement event was defined by pauses, reversals, or the length of the movie. Images (512x512 pixels) were acquired at a constant frame rate of 3.1 frames/s for a total of 500 frames. Image analysis was performed using ImageJ software, version 1.37 (National Institutes of Health, Bethesda, MD), and statistical significance was determined using the Student’s *t* test.

### Behavioral assays

The reversal frequency of individual animals was assayed as previously described [29]. Single young adult hermaphrodites were placed on NGM plates in the absence of food. The animals were allowed to adjust to the plates for 5 minutes, and the number of spontaneous reversals for each animal was counted over a 5-minute period. Twenty animals were tested for each genotype, and the reported scores reflect the mean number of reversals per minute.

### RNAi knockdown *rab-6*.*1* by hairpin RNA in the command interneurons

To perform *rab-6*.*1* RNAi in interneurons, we introduced a transgene that expresses a *rab-6*.*1* hairpin RNA into nematodes by germline transformation. First, we generated a transgene that contains head-to-head sense and antisense *rab-6*.*1* cDNAs into the *glr-1* promoter vector pV6. The vector pV6 has four restriction enzyme sites (SmaI, NheI, KpnI and SacI, ordered from 5’ to 3’ relative to the *glr-1* promoter) in its polycloning site downstream of the *glr-1* promoter. The second intron from gene *k02e10*.*1*, containing both 5’ and 3’ splicing sites (GT and AG), and 5–6 bp of flanking DNA was inserted into pV6 between NheI and KpnI to maximize transcript expression. Next, sequences encoding the complete RAB-6.1 ORF were inserted into the SmaI and NheI sites. Then the same sequences but in the reverse orientation were inserted into KpnI and SacI sites. The final construct was introduced into nematodes by germline transformation, with a concentration of 50 ng/μl plus 50 ng/μl coinjection marker *ttx-3*::*rfp*. The efficiency of RNAi was monitored by checking the fluorescent intensity of GFP::RAB-6.1 protein produced in nematodes harboring the transgene *P*_*glr-1*_::*gfp*::*rab-6*.*1*.

### Feeding RNAi

In intestinal cells, *rab-6*.*1* RNAi was performed by the feeding method [60]. *E*. *coli* strain HT115 carrying RNAi construct L4440-*rab-6*.*1* was cultured in 6 ml of LB medium containing 100 μg/ml ampicillin at 37°C overnight. The next day the cells were collected by centrifugation and resuspended in 400 μl of LB medium containing 100 μg/ml ampicillin, then seed onto four RNAi plates and left at room temperature for 1–2 days to air dry. Twenty adult animals were picked onto each RNAi plate and allowed to lay eggs for 2 hours, and then removed. Phenotypes were scored when F2 animals reached the adult or tested stage.

### Quantified laser scanning confocal microscopy

Autofluorescence from *C*. *elegans* intestinal gut granules interferes with the GFP signal detected at the 510 nm GFP emission peak. To obtain images of GFP fluorescence without interference from autofluorescence, we used the wavelength (lambda) scan function on the Leica TCS SP5 II confocal microscope (Leica Microsystems). Most confocal images were taken from nematodes at the L4 stage. Animals were mounted on 2% agarose pads with 10 mM levamisole at room temperature. A 63 × oil (NA 1.3) immersion objective (Leica TCS SP5 II) was used to detect fluorescent signals. Fluorescence profiles were drawn from signals recorded between 500 nm to 580 nm at 10 nm intervals. The GFP fluorescent signal was determined by comparing recorded profiles to reference autofluorescent profiles recorded from N2 worms. Autofluorescent signals were filtered out using the differences between these profiles. The quantification of intestinal fluorescent objects (i.e., MIG-14::GFP puncta) was done using ImageJ to automatically threshold the images and then measure integrated intensity of fluorescent objects within the region of interest (ROI) outlines defined by a 50 pixel radius circle.

## Supporting Information

S1 Text Sequence alignment of Rab6 orthologs. Text file of sequence alignments based on ClustalW for four human (H.s.) Rab6 isoforms, the yeast (S.c.) isoform Ypt6, and the two *C*. *elegans* (C.e.) Rab6 isoforms. Conservation of identical and similar amino acids are indicated by black and gray highlighting, respectively. “RabF” refers to Rab family specific regions. “RabSF” refers to Rab subfamily specific regions. “PM” refers to phosphate/magnesium binding residues. “G” refers to guanine nucleotide binding regions. “!” refers to amino acids that differentiate the Rab6A/RAB-6.1 subfamily from the Rab6B/RAB-6.2 subfamily. Orange asterisks indicate the three residues that differ between RAB6A and RAB6A’. Blue asterisks indicate the sites commonly mutated to generate GTP-locked and GDP-locked mutant proteins. The Switch I and Switch II regions are also indicated. Accession numbers: CAG46781.1 (Rab6A); AAF73841.1 (Rab6A’); AAF61637.1 (Rab6B); CAG38500.1 (Rab6C); CAA77590.1 (RAB-6.1); C*CD74453*.*1 (RAB-6*.*2); and Q99260*.*1 (Ypt6)*.
(DOCX)
