# Supplementary material for: RAB-6.1 and RAB-6.2 Promote Retrograde Transport in C. elegans
Source: PLoS One. 2016 Feb 18;11(2):e0149314. doi: 10.1371/journal.pone.0149314 (PMC4758642; doi:10.1371/journal.pone.0149314)
Supplement: S1 Text — Text file of sequence alignments based on ClustalW for four human (H.s.) Rab6 isoforms, the yeast (S.c.) isoform Ypt6, and the two C. elegans (C.e.) Rab6 isoforms. Conservation of identical and similar amino acids are indicated by black and gray highlighting, respectively. “RabF” refers to Rab family specific regions. “RabSF” refers to Rab subfamily specific regions. “PM” refers to phosphate/magnesium binding residues. “G” refers to guanine nucleotide binding regions. “!” refers to amino acids that differentiate the Rab6A/RAB-6.1 subfamily from the Rab6B/RAB-6.2 subfamily. Orange asterisks indicate the three residues that differ between RAB6A and RAB6A’. Blue asterisks indicate the sites commonly mutated to generate GTP-locked and GDP-locked mutant proteins. The Switch I and Switch II regions are also indicated. Accession numbers: CAG46781.1 (Rab6A); AAF73841.1 (Rab6A’); AAF61637.1 (Rab6B); CAG38500.1 (Rab6C); CAA77590.1 (RAB-6.1); CCD74453.1 (RAB-6.2); and Q99260.1 (Ypt6). (DOCX) [file pone.0149314.s001.docx]

**Switch I**

**G1** **PM2** **Switch II**

**RabSF1**  **PM1 * RabSF2**  **RabF1** ***RabF2** **PM3*** **RabF3**

*H.s.* RAB6A’ MSTGGDFGN-PLRKFKLVFLGEQSVGKTSLITRFMYDSFDNTYQATIGIDFLSKTMYLEDRTIRLQLWDTAGQERFRSLI
*H.s.* RAB6C MSAGGDFGN-PLRKFKLVFLGEQSVAKTSLITRFRYDSFDNTYQAIIGIDFLSKTMYLEDGTIGLRLWDTAGQERLRSLI
*H.s.* RAB6A MSTGGDFGN-PLRKFKLVFLGEQSVGKTSLITRFMYDSFDNTYQATIGIDFLSKTMYLEDRTVRLQLWDTAGQERFRSLI
*H.s.* RAB6B MSAGGDFGN-PLRKFKLVFLGEQSVGKTSLITRFMYDSFDNTYQATIGIDFLSKTMYLEDRTVRLQLWDTAGQERFRSLI
*C.e.* RAB-6.2 MS---DFGN-PLKKFKLVFLGEQSVGKTSLITRFMYDSFDNTYQATIGIDFLSKTMYLEDRTVRLQLWDTAGQERFRSLI
*C.e.* RAB-6.1 MA---DFTNNALKKFKLVFLGEQSVGKTSIITRFMYDSFDNTYQATIGIDFLSKTMYLEDRTIRLQLWDTAGQERFRSLI
*S.s.* Ypt6 MS---RSGK-SLTKYKIVFLGEQGVGKTSLITRFMYDTFDDHYQATIGIDFLSKTMYLDDKTIRLQLWDTAGQERFRSLI
 . | . | . | . | . | . | . | . |

10 20 30 40 50 60 70 80

**Switch II**

**RabF4**** **RabF5**  **! RabSF3 G2**  **!** **G3**

*H.s.* RAB6A’ PSYIRDSAAAVVVYDITNVNSFQQTTKWIDDVRTERGSD-VIIMLVGNKTDLADKRQVSIEEGERKAKELN-VMFIETSA
*H.s.* RAB6C PRYIRDSAAAVVVYDITNVNSFQQTTKWIDDVRTERGSD-VIITLVGNRTDLADKRQVSVEEGERKAKGLN-VTFIETRA
*H.s.* RAB6A PSYIRDSTVAVVVYDITNVNSFQQTTKWIDDVRTERGSD-VIIMLVGNKTDLADKRQVSIEEGERKAKELN-VMFIETSA
*H.s.* RAB6B PSYIRDSTVAVVVYDITNLNSFQQTSKWIDDVRTERGSD-VIIMLVGNKTDLADKRQITIEEGEQRAKELS-VMFIETSA
*C.e.* RAB-6.2 PSYIRDSTVAVVVYDITNSNSFHQTSKWIDDVRTERGSD-VIIMLVGNKTDLSDKRQVTTDEGERKAKELN-VMFIETSA
*C.e.* RAB-6.1 PSYIRDSSVAVVVYDITNANSFHQTTKWVDDVRNERGCD-VIIVLVGNKTDLADKRQVSTEDGEKKARDLN-VMFIETSA
*S.s.* Ypt6 PSYIRDSRVAIIVYDITKRKSFEYIDKWIEDVKNERGDENVILCIVGNKSDLSDERQISTEEGEKKAKLLGAKIFMETST
 . | . | . | . | . | . | . | . |

90 100 110 120 130 140 150 160

**G3** **RabSF4**

*H.s.* RAB6A’ KAGYNVKQLFRRVAAALPGMESTQDR--SREDMIDIKLEKP------QEQPVSEGGCSC
*H.s.* RAB6C KAGYNVKQLFRRVAAALPGMESTQDG--SREDMSDIKLEKP------QEQTVSEGGCSC
*H.s.* RAB6A KAGYNVKQLFRRVAAALPGMESTQDR--SREDMIDIKLEKP------QEQPVSEGGCSC
*H.s.* RAB6B KTGYNVKQLFRRVASALPGMENVQEK--SKEGMIDIKLDKP------QEPPASEGGCSC
*C.e.* RAB-6.2 KAGYNVKQLFRRIAGALPGIIKDDPV--EPPNVVTMDPIRQ------RQIVTDEGSCWC
*C.e.* RAB-6.1 KAGYNVKQLFRKIATALPGIVQEETP--EQPNIVIMNPPKD------AEESQGR-QCPC
*S.s.* Ypt6 KAGYNVKALFKKIAKSLPEFQNSESTPLDSENANSANQNKPGVIDISTAEEQEQSACQC
 . | . | . | . | . | .

170 180 190 200 210 219
